# Tumor Uptake of Triazine Dendrimers Decorated with Four, Sixteen, and Sixty-Four PSMA-Targeted Ligands: Passive versus Active Tumor Targeting

**DOI:** 10.3390/biom9090421

**Published:** 2019-08-28

**Authors:** Jongdoo Lim, Bing Guan, Kien Nham, Guiyang Hao, Xiankai Sun, Eric E. Simanek

**Affiliations:** 1 Department of Chemistry, Texas Christian University, Fort Worth, TX 76129, USA; 2 Department of Radiology, University of Texas Southwestern Medical Center, Dallas, TX 75390, USA; 3 Department of Urology, First Affiliated Hospital of Xi’an Jiaotong University, Xi’an 710043, China; 4 Advanced Imaging Research Center, University of Texas Southwestern Medical Center, Dallas, TX 75390, USA

**Keywords:** dendrimer, triazine, tumor, prostate-specific membrane antigen (PSMA), DUPA, PET Imaging, PC3-PIP, PC3-FLU, CT, EPR, ^64^Cu, prostate cancer

## Abstract

Various glutamate urea ligands have displayed high affinities to prostate specific membrane antigen (PSMA), which is highly overexpressed in prostate and other cancer sites. The multivalent versions of small PSMA-targeted molecules are known to be even more efficiently bound to the receptor. Here, we employ a well-known urea-based ligand, 2-[3-(1,3-dicarboxypropyl)-ureido] pentanedioic acid (DUPA) and triazine dendrimers in order to study the effect of molecular size on multivalent targeting in prostate cancer. The synthetic route starts with the preparation of a dichlorotriazine bearing DUPA in 67% overall yield over five steps. This dichlorotriazine reacts with G1, G3, and G5 triazine dendrimers bearing a 1,4,7,10-tetraazacyclododecane-1,4,7,10-tetraacetic acid (DOTA) group for ^64^Cu-labeling at the core to afford poly(monochlorotriazine) intermediates. Addition of 4-aminomethylpiperidine (4-AMP) and the following deprotection produce the target compounds, G1-(DUPA)_4_, G3-(DUPA)_16_, and G5-(DUPA)_64_. These targets include 4/16/64 DUPA groups on the surface and a DOTA group at the core, respectively. In vitro cell assay using PC3-PIP (PSMA positive) and PC3-FLU (PSMA negative) cells reveals that G1-(DUPA)_4_ has the highest PC3-PIP to PC3-FLU uptake ratio (10-fold) through the PSMA-mediated specific uptake. While G5-(DUPA)_64_ displayed approximately 12 times higher binding affinity (IC_50_ 23.6 nM) to PC3-PIP cells than G1-(DUPA)_4_ (IC_50_ 282.3 nM) as evaluated in a competitive binding assay, the G5 dendrimer also showed high non-specific binding to PC3-FLU cells. In vivo uptake of the ^64^Cu-labeled dendrimers was also evaluated in severe combined inmmunodeficient (SCID) mice bearing PC3-PIP and PC3-FLU xenografts on each shoulder, respectively. Interestingly, quantitative imaging analysis of positron emission tomograph (PET) displayed the lowest tumor uptake in PC3-PIP cells for the midsize dendrimer G3-(DUPA)_16_ (19.4 kDa) (0.66 ± 0.15%ID/g at 1 h. p.i., 0.64 ± 0.11%ID/g at 4 h. p.i., and 0.67 ± 0.08%ID/g at 24 h. p.i.). Through the specific binding of G1-(DUPA)_4_ to PSMA, the smallest dendrimer (5.1 kDa) demonstrated the highest PC3-PIP to muscle and PC3-PIP to PC3-FLU uptake ratios (17.7 ± 5.5 and 6.7 ± 3.0 at 4 h p.i., respectively). In addition, the enhanced permeability and retention (EPR) effect appeared to be an overwhelming factor for tumor uptake of the largest dendrimer G5-(DUPA)_64_ as the uptake was at a similar level irrelevant to the PSMA expression.

## 1. Introduction

Targeted delivery is one of the key elements in the design of new drugs as it can improve their safety and efficacy by reducing the nonspecific uptake-associated toxicity, increasing the maximum tolerated dose (MTD), and enhancing the cellular uptake in specific disease sites [1,2]. When combined with imaging probes, the targeted drug delivery also affords a critical tool to evaluate the diseases noninvasively during the treatment with high sensitivity and specificity. Elevated cellular drug uptake by the targeted diseased cells can be actively obtained from the proper selection of a disease-specific receptor, a targeting ligand, and a spacer [1,3]. Furthermore, nano-sized drugs (typically, MW 40–800 kDa) can be passively accumulated in tumor tissues via the enhanced permeability and retention (EPR) effect [4] due to the unique pathophysiological facets found in most solid tumors such as defective vasculature and impaired lymphatic drainage.

Prostate cancer is the second most common cancer among men in the United States with 164,690 estimated new cases and 29,430 estimated deaths in 2018 [5]. Various cell surface markers have been evaluated for diagnosis and treatment of patients suffering from prostate cancer, including prostate specific antigen (PSA) [6], prostatic acid phosphatase (PAP) [7], prostate stem cell antigen (PSCA) [8], and prostate specific membrane antigen (PSMA) [9,10]. Among them, PSMA (also known as glutamate carboxypeptidase II), a type II transmembrane glycoprotein has a significant potential for both imaging and therapeutics in prostate cancer as it is upregulated 100~1000-fold higher in prostate cancer cells compared to healthy prostate cells and even more expressed in aggressive/metastatic cancers [11,12,13]. Therefore, PSMA-targeting agents including aptamers [14,15], antibodies [16], peptides [17], and small molecule inhibitors [18] have been extensively studied and applied to small molecule drug conjugates [19,20,21] and nanomedicines [22,23,24,25] for diagnosis and treatment in prostate cancer.

Diverse urea-based small molecules have been identified as promising PSMA-targeting ligands. Since *N*-acetyl-L-aspartyl-L-glutamate (NAAG) in PSMA is enzymatically hydrolyzed to *N*-acetyl-L-aspartate and glutamate by *N*-acetylated-α-linked-acidic dipeptidase (NAALADase), the NAAG analogs may possess high affinity to PSMA inhibiting the enzymatic activity of NAALADases [26]. A synthetic urea-based ligand, 2-[3-(1,3-dicarboxypropyl)-ureido] pentanedioic acid (DUPA) is known to selectively bind to PSMA with a high affinity (*K*i = 8.0 nM, IC_50_ = 47 nM) [27,28] and has been successfully employed to PET imaging, therapeutics, and theranostics in prostate cancers [18,29,30,31]. In addition, the multivalent scaffolds with glutamate urea ligands displayed even higher affinity to PSMA with approximately 3- to 10-fold higher inhibitory activity compared to the monovalent counterparts [22,24,32,33,34,35,36,37,38,39,40].

Of the dendrimers that have been explored as platforms for multivalent display of PSMA-targeting ligands, two examples are noteworthy. First, a hexavalent platform based on a generation one dendrimer, which incorporated chelating groups into the backbone, showed notably enhanced affinity for LMCaP tumors [40]. Second, a generation 5 PAMAM dendrimer-methotrexate conjugate bearing multiple copies of glutamate urea (GLA) was evaluated with PSMA positive LNCaP cells in vitro and displayed elevated inhibition of the NAALADase activity when compared with free GLA at equimolar concentrations [41]. With increasing size of drug conjugates, however, targeted drug delivery to tumor cells is more complicated as the EPR effect can affect the biodistribution and tumor localization [42]. For instance, a class of gold nanorods with multiple targeting peptides only slightly enhanced the tumor uptake in tumor xenograft models compared with non-targeted controls [43]. The tumor uptake level of long-circulating anti-HER2 immunoliposomes was also similar to that of nontargeted liposome counterparts [44]. On the contrary, a recombinant protein of human serum albumin (HSA, 67 kDa) was localized up to three times more in a tumor over time when fused with a tumor targeting agent, amino-terminal fragment (ATF) [45]. To date, it has been largely unclear how the interplay of the molecular size, multivalency, and receptor-binding affinity of nanomedicines affects their in vivo kinetics and targeted uptake.

**Chart 1:**
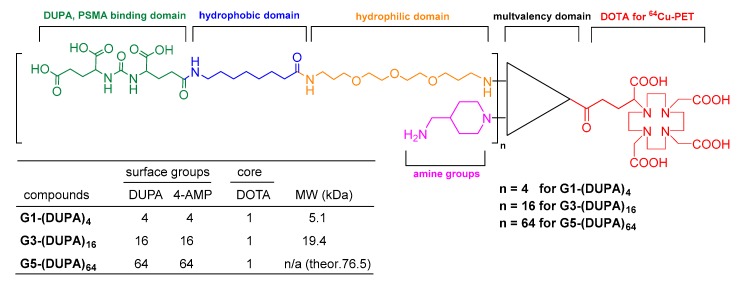
Schematic Design of Triazine Dendrimers bearing DUPA ligands on the Surface and a DOTA at the Core.

Triazine dendrimers have been shown to be great platforms for drug delivery due to the well-defined (often monodisperse) globular structures, multivalency, and facile synthesis. The target molecules here are designed and synthesized using a combined strategy that employs odd generations of triazine dendrimers and a dichlorotrizine (DCT) functionalized monomer (Chart 1). The odd generation dendrimers (G1, G3, and G5) are prepared using an iterative two-reactions-per-generation from a triazine core with a ^64^Cu-labeling DOTA [46,47]. A dichlorotriazine bearing a DUPA ligand includes both hydrophobic and hydrophilic chains that may help bind to the deep hydrophobic pocket of PSMA [31]. The conjugation of dendrimer with DCT affords poly(monochlorotriazine), which is subsequently capped with 4-aminomethylpiperidine (4-AMP). Following deprotection, the target compounds, G1-(DUPA)_4_, G3-(DUPA)_16_, and G5-(DUPA)_64_ include 4/16/64 of DUPA ligands for binding to PSMA, 4/16/64 of 4-AMP groups for post-modification with therapeutic moiety, and a DOTA for ^64^Cu-labeling. Tumor uptake of the dendrimers in a mouse model of prostate cancer is evaluated as well as in vitro cell assay using PSMA positive/negative cell lines. Accordingly, we discuss the relative contribution of active/passive targeting for tumor localization depending on the number of ligands and the size of dendrimers.

## 2. Materials and Methods

### 2.1. General Procedures

Macromonomeric MCT [46], Triazine Core [46], and ^125^I-ligand 7 [21] were synthesized as previously reported. DUPA-tris (t-Butyl ester) was also prepared as previously reported [28]. DOTAGA-tetra (t-Bu ester) was purchased from Macrocyclics. Other chemicals were purchased from Sigma-Aldrich, Acros, and Chem-Impex International. All organic solvents were ACS/HPLC grade and used without further purification. All aqueous solutions were prepared using Milli-Q water (18 MΩ-cm) obtained from a Millipore Gradient Milli-Q water system. NMR spectra were recorded on a Bruker Ascend 400 MHz spectrometer in CDCl_3_ or CD_3_OD. All mass spectral analyses were carried out by an Agilent Technologies 6224 TOF LC/MS system. Radio labeling was evaluated by a Waters 600 HPLC system equipped with a Waters 2996 photodiode array detector, an in-line Shell Jr. 2000 radiodetector, and a Waters Atlantis T3 column (5 µm, 4.6 × 250 mm). The mobile phase consisted of water/acetonitrile (60/40, HPLC grade, 0.1% (*v*/*v*) trifluoroacetic acid) at a flow rate of 1 mL/min.

### 2.2. Synthesis

The experimental details for synthesis and spectra are found in the Supporting Information.

### 2.3. Cell Lines and Animal Models

The PC3-PIP and PC3-FLU cell lines were obtained from Dr. Martin G. Pomper of John Hopkins University. The cell lines PC3-PIP and PC3-FLU were cultured in RPMI media supplemented with 10% fetal bovine serum (FBS), 1% penicillin/streptomycin, and 2 µg/mL of puromycin. After cultured at 37 °C in an atmosphere of 5% CO_2_ and passaged at 75% to 90% confluency, the cell lines were harvested using PBS and trypsin, and suspended in RPMI media with 5% FBS. The suspension of PC3-PIP (2 × 10^6^) in 100 µL of media was subcutaneously injected into male SCID mice over the left shoulder. The PC3-FLU (2 × 10^6^) cells was also subcutaneously injected into the same mouse over the right shoulder. The PET/CT imaging studies were performed using mice with the tumor size range from 40 to 800 mm^3^ (tumor volume = ½ (length × width^2^). All animal studies were approved by the Institutional Animal Care and Use Committee at UT Southwestern (IACUC; APN 2017-102312).

### 2.4. Preparation of ^64^Cu-Labeld G1-(DUPA)_4_, G3-(DUPA)_16_, and G5-(DUPA)_64_

A solution of each dendrimer in PBS (pH 7.4) was added to a solution of ^64^CuCl_2_ in PBS with a 37 MBq/nmol concentration. The resulting solution was reacted at 75 °C for 1–2 h. After addition of 5 mM EDTA (100-fold molar excess), the solution was incubated at 37 °C for 10 min and monitored by radio-TLC. The ^64^Cu-labeled agents were purified using an Amicon Ultra centrifugal filters (MWCO 5 k, 10 k, and 30 k; 5000× *g*). The purified agents were developed in a solution of 5 mM EDTA/PBS (1/1) solution with instant thin-layer chromatography (ITLC-SG) plates and analyzed by a Rita Star Radioisotope TLC Analyzer. The radiochemical purity of ^64^Cu-labeled agents showed >97% as determined by radio-HPLC and radio-TLC.

### 2.5. Serum Stability Assay

The stability of ^64^Cu-labeled dendrimers was evaluated in rat serum. Each of ^64^Cu-labeled dendrimers (0.5 MBq, 10 µL) were added to a suspension of 90 µL of rat serum and incubated at 37 °C for 1, 4, and 24 h. An aliquot of sample (10 µL) was mixed with 5 mM EDTA (100-fold molar excess) and incubated at 37 °C for 10 min. The resulting solution was analyzed by a Rita Star Radioisotope TLC Analyzer.

### 2.6. Competition Assay

A competitive cell-binding assay of G1-(DUPA)_4_, G3-(DUPA)_16_, and G5-(DUPA)_64_ was performed to evaluate PSMA binding affinity. A reported PSMA ^125^I-radioligand [21] was used as a PSMA-specific radioligand in the assay. PC3-PIP cells were suspended in Tris-buffered saline (TBS) and seeded on multiwell DV plates (Millipore) with 5 × 10^4^ cells per well. The seeded wells (200 µL) were then incubated at room temperature for 2 h (*n* = 4) with the ^125^I-radioligand (33,000 cpm/well) in the presence of increasing concentrations (0−2500 nM) of G1-(DUPA)_4_, G3-(DUPA)_16_, and G5-(DUPA)_64_. The unbound ^125^I-radioligand was removed by filtration. The wells were rinsed five times with cold TBS buffer. The filters were then collected and their radioactivity was measured on a 2480 automatic gamma counter (PerkinElmer). The best-fit IC_50_ values were calculated for G1-(DUPA)_4_, G3-(DUPA)_16_, and G5-(DUPA)_64_ by fitting the data with nonlinear regression using GraphPad Prism 6.0.

### 2.7. Cell Uptake and Internalization

Cell uptake experiments were conducted in the PSMA positive PC3-PIP cell lines and the PSMA negative PC-FLU cell lines. The cells (5.0 × 10^5^) were seeded to a 6-well plate and incubated at 37 °C overnight in a humidified incubator with 5% CO_2_. After being rinsed with binding buffer (20 mM Tris, 150 mM NaCl, pH 7.4), the cells in binding buffer (500 µL) were incubated at 37 °C with ~6.0 × 10^5^ CPM of ^64^Cu-labeled dendrimers for 1 h, washed with cold binding buffer, and then trypsinized. The radioactivity of trypsinized cells was counted on a 2480 automatic gamma counter (PerkinElmer).

The PC3-PIP cells were used for the internalization study. The 12-well plates containing ~2.0 × 10^5^ cells were incubated overnight in a humidified incubator at 37 °C with 5% CO_2_ and then washed with binding buffer (20 mM Tris, 150 mM NaCl, pH 7.4). Each solution of ^64^Cu-labeled dendrimers (~6.0 × 10^5^ CPM) in 0.4 mL of binding buffer was incubated with the cells for 1, 10, 30, 60, and 120 min, respectively, and then washed once with ice-cold binding buffer. The surface bound ^64^Cu-labeled dendrimers were removed by incubating cells twice for 5 min with 0.5 mL of ice-cold low pH stripping buffer (150 mM NaCl, 50 mM glycine, pH 3.0) and collected in a culture tube. After incubation with 0.5 mL of 4 M NaOH at 37 °C for 15 min, the NaOH-solubilized cells were collected in separate culture tubes. The radioactivity of surface bound/internalized ^64^Cu-labeled dendrimers was counted on a 2480 automatic gamma counter (PerkinElmer).

### 2.8. Small Animal PET/CT Imaging

The PET/CT imaging studies were performed with a Siemens Inveon PET-CT Multimodality System (Siemens Medical Solutions Inc., Knoxville, TN, USA). Each male SCID mouse bearing PC3-PIP (PSMA positive, left shoulder) and PC3-FLU (PSMA negative, right shoulder) intravenously received about 3.7 MBq of ^64^Cu-labeled dendrimers (100 µL in PBS) via the tail vein (*n* = 3 for G1-(DUPA)_4_ and G5-(DUPA)_64_; *n* = 4 for G3-(DUPA)_16_). The mouse was sedated on the imaging bed using 1–2% isofluorane anesthesia for the duration of imaging. Static PET scans were conducted for 15 min at 1, 4, and 24 h post injection (p.i.) and then followed by a CT scan (7 min). The obtained PET and CT images were reconstructed and fused for quantitative data analysis.

## 3. Results and Discussion

### 3.1. Synthesis and Characterization of G1-(DUPA)_4_, G3-(DUPA)_16_, and G5-(DUPA)_64_

**Scheme 1:**
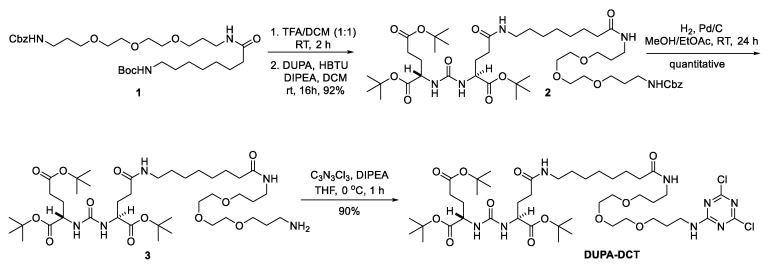
Synthesis of DUPA-DCT.

**Scheme 2:**
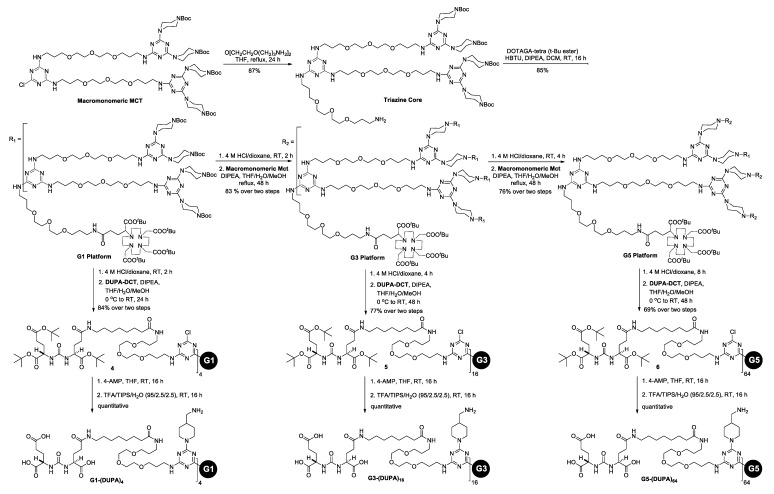
Synthesis of G1-(DUPA)_4_, G3-(DUPA)_16_, and G5-(DUPA)_64_.

The triazine dendrimers bearing DUPA ligands were synthesized using a similar strategy as previously published [46,47,48]. The synthetic route of dichlorotriazine intermediate (DUPA-DCT) is described in Scheme 1. The synthesis starts with the preparation of protected diamine **1** by coupling reaction of boc-8-aminocaprylic acid with *N*-Cbz-4,7,10-trioxa-1,13-tridecanediamine using 1,1’-carbonyldiimidazole. Following the BOC deprotection, the amine was coupled with DUPA-tris (t-Butyl ester) using HBTU as a coupling agent to provide **2** in 92% yield. The cbz-amine was unmasked by hydrogenation and followed by reaction with cyanuric chloride to afford DUPA-DCT. DUPA-DCT was prepared in 67% overall yield over five steps. The preparation of dendrimer platforms with a DOTA at the core and the installation of DUPA groups on the surface are shown in Scheme 2. Following reaction of Macromomeric MCT with a flexible diamine, the DOTA group for ^64^Cu-labeling was installed on Triazine Core using HBTU. The iterative deprotection and macromomer addition provided the larger generation platforms (G3 and G5). The poly(monochlorotriazine) intermediates, **4**, **5**, and **6** were accessed upon reaction of DUPA-DCT with the deprotected G1/G3/G5 platforms, respectively. The monochlorotriazine groups were then capped with 4-aminomethylpiperidine (4-AMP). The following deprotection in TFA/TIPS/H_2_O (95/2.5/2.5) afforded G1-(DUPA)_4_, G3-(DUPA)_16_, and G5-(DUPA)_64_. The final compounds, G1-(DUPA)_4_, G3-(DUPA)_16_, and G5-(DUPA)_64_ were synthesized from Triazine Core in 71%, 54%, and 37% overall yields over five, seven, or nine linear steps, respectively.

**Figure 1:**
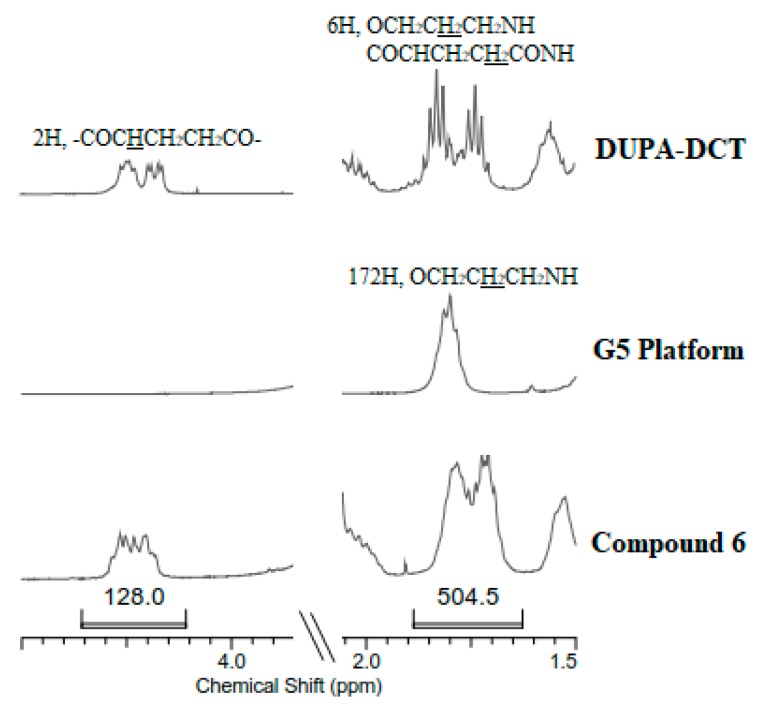
^1^H NMR spectra of DUPA-DCT, G5 Platform, and 6 in the distinctive region for monitoring reaction of the DCT with the deprotected G5 dendrimer. The theoretical integration value of 6, which appeared at 1.75 ppm, is supposed to be 556.

The synthesis and characterization are followed by NMR spectroscopy, mass spectrometry, and thin layer chromatography. G1 dendrimer and small intermediates are readily characterized and confirmed by the common techniques above. As generation increases, however, NMR spectroscopy is often less useful as repeating units give overwhelming signals subduing other signals. For instance, the NMR signals from DOTA at the core of G3 and G5 were too weak to give reliable signal-to-noise ratios. Mass spectrometry is very practical to confirm the desired products and to detect the by-products of incomplete substitution. The targets and intermediates of G1 and G3 were shown to be single entity by ESI-TOF mass spectrometry. However, the G5 poly(monochlorotriazine) intermediate **6** failed to give mass ion species as it occasionally occurred with large dendrimers. Nucleophilic aromatic substitution of the DCT intermediate with the deprotected G5 dendrimers is a critical step to afford monodisperse entities or low dispersity mixtures, which is important for reproducible pharmacokinetics and pharmacodynamics. NMR spectroscopy is still a useful tool to characterize **6**, despite the limitations described above. As shown in Figure 1, we followed substitution of the DCT with 64 piperazine groups of G5 using ^1^H NMR spectroscopy. Most signals of the ^1^H NMR spectra were highly overlapped, making integration values less reliable. With a total of 64 substituted DUPA-DCT groups, the peaks of poly(monochlorotriazine) **6** at 4.25 and 1.75 ppm give the theoretical integrated values, 128 and 556, respectively. The actual integration value at 1.75 ppm appeared to be 504.5 that is off by 51.5 (9%). As the integration in ^1^H NMR spectroscopy typically displays accuracy of ± 5%, the 9% appears a little high. However, considering the elevated inaccuracy of integration found for large molecules in this mass range, the defects derived from incomplete reaction appear to be negligible if exist.

### 3.2. Radiolabeling and Serum Stability

^64^Cu-Labeling yield was achieved in >80% in 1 h for both G1-(DUPA)_4_ and G3-(DUPA)_16_. The radiolabeling of G5-(DUPA)_64_ had a lower radiochemical yield of 50% and also needed a longer incubation time (2 h) as the radiometal becomes less accessible to the DOTA at the core of higher generations. The radiochemical purity of the ^64^Cu-labeled dendrimers was shown to be >97% as determined by radio-TLC and radio-HPLC. The serum stability was performed by incubating the ^64^Cu-labeled dendrimers in rat serum at 37 °C for 1, 4, and 24 h. The incubation solution was then challenged with EDTA (100-fold molar excess) at 37 °C for 10 min. The ^64^Cu labeled G1-(DUPA)_4_ and G3-(DUPA)_16_ were stable in rat serum and remained intact over 24 h, while the radio-labeled G5-(DUPA)_64_ displayed a little shoulder peak (5% at 4 h; 10% at 24 h) as shown in radio-TLC (Figure S41). As ^64^Cu-EDTA moves to the solvent front when developed in the employed TLC condition, this new shoulder peak could be derived from the binding between serum protein and the ^64^Cu-labeld dendrimer. Also, Diallo et al. described how larger generations of PAMAM dendrimer displayed a higher binding constant for serum protein [49]. This indicates that the biodistribution of large dendrimers may be further influenced by such non-specific interaction and binding in vivo.

### 3.3. In Vitro Cell Assays

**Figure 2:**
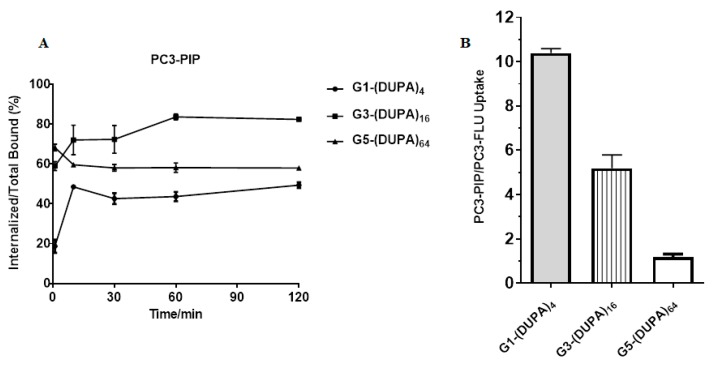
In vitro cell assays. (**A**) Internalization of the ^64^Cu-labeld dendrimers in PC3-PIP cells. (**B**) The cellular uptake ratio of the ^64^Cu-labeld dendrimers in PC3-PIP (PSMA^+^) and PC3-FLU (PSMA^−^) cells (*n* = 3).

The PSMA-mediated cellular uptake of the ^64^Cu-labeled dendrimers and the internalization were studied using PC3-PIP (PSMA positive) cells and PC3-FLU (PSMA negative) cells (Figure 2). G5-(DUPA)_64_ was immediately internalized to PC3-PIP reaching the maximum value 68% and the level of internalization remained similar over 120 min. The highest internalization of G3-(DUPA)_16_ was 84% at 60 min, whereas that of G1-(DUPA)_4_ was only 49%. All the dendrimers were shown to be quickly internalized, possibly due to multivalent DUPA ligands on the surface of dendrimers. The cellular uptake ratio of dendrimers was measured using PC3 PIP and PC3-FLU cells. As expected, the cellular uptake of G1-(DUPA)_4_ in PC3-PIP was 10 times higher than that in PC3-FLU by virtue of the PSMA-mediated binding. Interestingly, the PC3-PIP to PC3-FLU cell uptake ratios of G3-(DUPA)_16_ and G5-(DUPA)_64_ were lower: 5.2 ± 1.0 and 1.2 ± 0.2_,_ respectively. Thus, the larger generation dendrimer with the more DUPA ligands displayed higher non-specific cellular uptake. In fact, G5-(DUPA)_64_ displayed the highest cellular uptake in both PSMA negative and positive cells compared to G1-(DUPA)_4_ and G3-(DUPA)_16_. A certain extent of non-specific cellular uptake can be observed for highly multivalent dendrimers through passive endocytosis. However, the cause of this high non-specific uptake found for G5-(DUPA)_64_ is largely unclear, while electrostatic interaction between cationic AMP groups and anionic cell membrane might elevate non-specific uptake to some extent.

**Figure 3:**
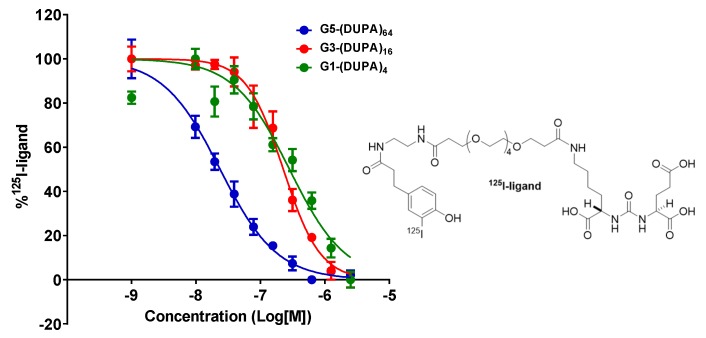
The PSMA binding affinities of G1-(DUPA)_4_, G3-(DUPA)_16_, and G5-(DUPA)_64_ were evaluated using PC3-PIP cells and a ^125^I-labeled PSMA radioligand as the PSMA-specific competitor. The IC_50_ values of G1-(DUPA)_4_, G3-(DUPA)_16_, and G5-(DUPA)_64_ were calculated to be 23.6 nM (*R^2^* = 0.95), 226.7 nM (*R^2^* = 0.94), and 282.3 nM (*R^2^* = 0.88), respectively (*n* = 4).

Competitive binding assay of the ^64^Cu-labeled dendrimers was performed in PC3-PIP cells (PSMA positive) using a reported PSMA ^125^I-labeled radioligand as the PSMA-binding competitor (Figure 3). The IC_50_ values of dendrimers were determined by the concentration of dendrimer displacing 50% of the ^125^I-labed PSMA-radioligand bound to PC3-PIP cells: 23.6 nM for G5-(DUPA)_64_, 226.7 nM for G3-(DUPA)_16_, and 282.3 nM for G1-(DUPA) _4_, respectively. These results indicate that the dendrimers with the more DUPA ligands contain the stronger binding affinity to PSMA in vitro.

### 3.4. PET/CT Imaging in Mouse Models

**Figure 4:**
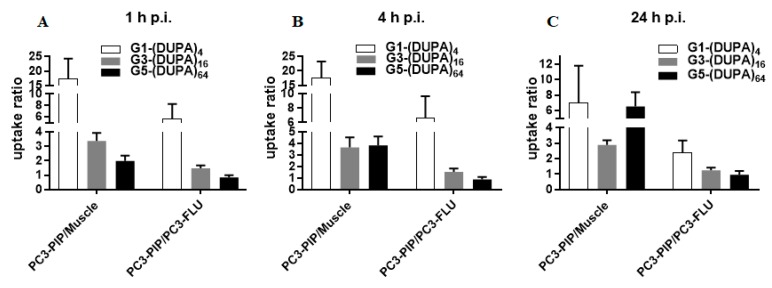
PC3-PIP to muscle and PC3-PIP to PC3-FLU uptake ratios of the ^64^Cu-labeled dendrimers in SCID mice bearing PC3-PIP cells on the left shoulder and PC3-FLU cells on the right shoulder at 1 h p.i. (**A**), 4 h p.i. (**B**), and 24 h p.i. (**C**). (*n* = 3 for G1-(DUPA)_4_ and G5-(DUPA)_64_; *n* = 4 for G3-(DUPA)_16_).

**Figure 5:**
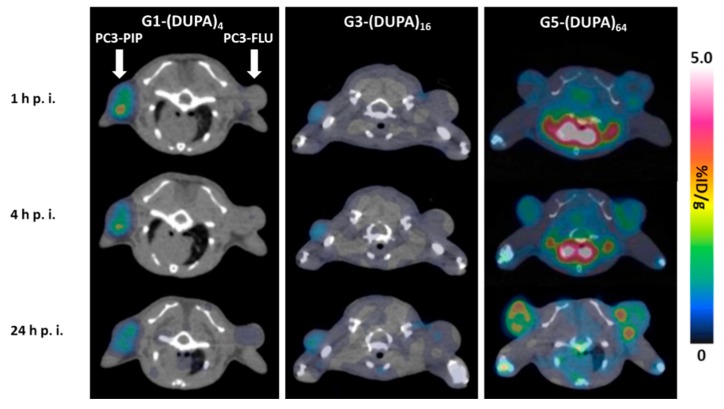
Representative trans-axial PET-CT images of the ^64^Cu-labeled dendrimers in SCID mice bearing PSMA^+^ PC3-PIP xenografts on the left shoulder and PSMA^-^PC3-FLU xenografts on the right shoulder at 1, 4, and 24 h p.i. (*n* = 3 for G1-(DUPA)_4_ and G5-(DUPA)_64_; *n* = 4 for G3-(DUPA)_16_).

In vivo uptake of the ^64^Cu-labeled dendrimer was evaluated using SCID mice bearing PC3-PIP (PSMA positive) xenografts on the left shoulder and PC3-FLU (PSMA negative) xenografts on the right shoulder (Table 1 and Figure 4). As summarized in Table 1, the smaller dendrimers, G1-(DUPA)_4_ and G3-(DUPA)_16_ displayed high kidney uptake through renal clearance and a subset of mouse proximal renal tubules [50]. The level of kidney uptake was consistent over 24 h p.i showing approximately 45% to55%ID/g for G1-(DUPA)_4_ and ~45%ID/g for G3-(DUPA)_16_, respectively. On the contrary, G5-(DUPA)_64_ exhibited much lower kidney uptake (3% to 6%ID/g) and was highly taken up by the liver via the reticuloendothelial systems (RES) (11.43 ± 1.27%ID/g at 1 h p.i., 13.67 ± 0.97%ID/g at 4 h p.i., and 18.90 ± 1.04%ID/g at 24 h p.i.). As expected, the high level of heart uptake was observed for G5-(DUPA)_64_ derived from prolonged blood circulation as such a large dendrimer (76.5 kDa) cannot be efficiently cleared by renal filtration. Interestingly, the midsize dendrimer G3-(DUPA)_16_ (19.4 kDa) displayed the lowest tumor uptake in PSMA positive PC3-PIP cells. This tumor uptake pattern is also found in targeting antibodies: Wittrup and Schimidt reported that intermediate-sized targeting antibodies (MW ~25 kDa) showed the lowest tumor uptake compared with smaller and larger targeting antibodies [42]. The tumor uptake of G5-(DUPA)_64_ increased throughout the 24-h period in both PC3-PIP and PC3-FLU cells possibly due to the EPR effect, whereas G1-(DUPA)_4_ reached the highest tumor uptake at 1 h p.i. in PC3-PIP xenografts via the PSMA-mediated tumor targeting (Table 1). As shown in Figure 4, the EPR effect appeared to be the dominant factor for tumor localization of the largest dendrimer G5-(DUPA)_64_. That is, irrespective of PSMA negative/positive cell lines, the uptake level was similar. Instead, G1-(DUPA)_4_ displayed the highest PC3-PIP to muscle and PC3-PIP to PC3-FLU uptake ratios (17.7 ± 5.5 and 6.7 ± 3.0 at 4 h p.i., respectively) indicating that the dendrimer was mainly taken up by the PSMA-specific binding. Such tumor localization is well observed in the representative trans-axial PET-CT images in Figure 5. G1-(DUPA)_4_ provides a clear target-to-background image of PC3-PIC xenografts on the left shoulder of mouse, while providing a very low target-to-background contrast image of PC3-FLU xenografts on the right shoulder. With a relatively high background radioactivity due to the longer retention time in the blood, G5-(DUPA)_64_ showed a similar PET/CT signal intensity in both PSMA positive/negative xenografts. As the EPR effect is known to be effective for a large molecule (> 40 kDa), G3-(DUPA)_16_ (19.4 kDa) was expected to display low EPR effect in tumor sites. However, the non-targeted tumor uptake was significantly elevated in G3-(DUPA)_16_ when compared with G1-(DUPA)_4_. Overall, the PSMA-specific uptake of the dendrimers bearing multiple copies of DUPA ligands (4, 16, and 64) decreases with increasing the molecular size regardless of the number of ligands.

## 4. Conclusions

In summary, we have designed and synthesized three dendrimers bearing 4/16/64 DUPA ligands on the surface and a DOTA chelator at the core. The synthesis started with the preparation of dichlorotriazine (DCT) intermediate including a DUPA ligand and G1/G3/G5 triazine dendrimer platforms including a DOTA group. Installation of the DCT intermediate on the dendrimer platforms, addition of 4-AMP, and the subsequent deprotection afforded the final products, G1-(DUPA)_4_, G3-(DUPA)_16_, and G5-(DUPA)_64_ with well-defined structure with dimensions of approximately 2, 4, and 8 nm as estimated by dynamic light scattering of unfunctionalized analogues [51]. Targeted/non-targeted uptake of the ^64^Cu-labeled dendrimers were systematically evaluated both in vitro and in vivo using PC3-PIP (PSMA positive) and PC3-FLU (PSMA negative) cells. In cell-based assays, G1-(DUPA)_4_ displayed the highest PC3-PIP to PC3-FLU uptake ratio. Although G5-(DUPA)_64_ displayed the highest binding affinity to PSMA, non-specific uptake of the dendrimer in PC3-FLU was also high. In vivo tumor uptake studies displayed that non-targeted uptake was elevated with increasing the molecular size of dendrimers despite the advantageous multivalency of larger dendrimers. This result indicates that the EPR effect contributes significantly to non-receptor-mediated uptake of large molecules regardless of the number of targeting ligands. In fact, the same level of targeted/non-targeted tumor uptake was found in G5-(DUPA)_64_ bearing 64 DUPA ligands, suggesting such uptake was dominated by the EPR effect.

## Figures and Tables

**Table 1:** In vivo organ uptake (%ID/g ± s.d.) of the ^64^Cu-labeled dendrimers in SCID mice bearing PC3-PIP cells on the left shoulder and PC3-FLU cells on the right shoulder at 1, 4, and 24 h p.i. (*n* = 3 for G1-(DUPA)_4_ and G5-(DUPA)_64_; *n* = 4 for G3-(DUPA)_16_)

	G1-(DUPA)_4_			G3-(DUPA)_16_			G5-(DUPA)_64_		
	1 h p.i.	4 h p.i.	24 h p.i.	1 h p.i.	4 h. p.i.	24 h. p.i.	1 h p.i.	4 h. p.i.	24 h. p.i.
Brain	0.03 ± 0.01	0.02 ± 0.00	0.07 ± 0.00	0.04 ± 0.01	0.04 ± 0.01	0.07 ± 0.03	0.94 ± 0.10	0.66 ± 0.08	0.20 ± 0.06
Heart	0.16 ± 0.05	0.10 ± 0.02	0.25 ± 0.05	0.20 ± 0.03	0.17 ± 0.03	0.29 ± 0.03	15.37 ± 1.46	10.77 ± 1.55	1.60 ± 0.10
Liver	1.03 ± 0.16	1.20 ± 0.20	1.93 ± 0.15	1.23 ± 0.21	1.45 ± 0.13	2,20 ± 0.27	11.43 ± 1.27	13.67 ± 0.97	18.90 ± 1.04
Left Kidney	53.27 ± 4.37	58.73 ± 4.98	54.00 ± 5.96	42.85 ± 3.43	45.00 ± 4.63	43.85 ± 5.13	6.10 ± 1.57	5.47 ± 0.76	2.93 ± 0.59
Right Kidney	56.83 ± 5.14	59.97 ± 3.27	55.03 ± 7.36	44.28 ± 4.79	48.55 ± 6.59	46.95 ± 6.35	6.23 ± 0.29	5.73 ± 0.51	3.17 ± 0.45
Muscle	0.06 ± 0.03	0.05 ± 0.01	0.13 ± 0.03	0.20 ± 0.04	0.18 ± 0.02	0.23 ± 0.03	0.49 ± 0.07	0.42 ± 0.06	0.29 ± 0.06
Bone	0.08 ± 0.03	0.07 ± 0.01	0.08 ± 0.02	0.27 ± 0.08	0.22 ± 0.03	0.27 ± 0.08	0.93 ± 0.04	0.90 ± 0.17	0.98 ± 0.20
PC3-PIP	0.99 ± 0.36	0.87 ± 0.40	0.81 ± 0.36	0.66 ± 0.15	0.64 ± 0.11	0.67 ± 0.08	0.97 ± 0.03	1.57 ± 0.15	1.83 ± 0.21
PC3-FLU	0.18 ± 0.05	0.13 ± 0.02	0.33 ± 0.06	0.45 ± 0.06	0.42 ± 0.05	0.54 ± 0.08	1.16 ± 0.21	1.83 ± 0.32	1.97 ± 0.31

## Supplementary Materials

The following are available online at https://www.mdpi.com/2218-273X/9/9/421/s1. Synthetic procedures, Compound Spectra, Radio-HPLC data and Serum Stability Assays.
