# Supplementary material for: Tumor Uptake of Triazine Dendrimers Decorated with Four, Sixteen, and Sixty-Four PSMA-Targeted Ligands: Passive versus Active Tumor Targeting
Source: Biomolecules. 2019 Aug 28;9(9):421. doi: 10.3390/biom9090421 (PMC6770530; doi:10.3390/biom9090421)
Supplement: Supplementary file 1 [file biomolecules-09-00421-s001.pdf]

Supporting Information for

Tumor Uptake in Triazine Dendrimers Decorated with Four, Sixteen, and Sixty-Four PSMA-Targeted Ligands: Passive versus Active Tumor Targeting

Table of Contents

Synthetic Procedures…………………………………………………………………..…..….S2

Spectra………………………………………………………………………………….….S17

Radio-HPLC………………………………………………………………………………S41

Serum Stability Assay ……………………………………………………………...…….S42

Synthetic Procedures

Compound 1. Boc-8-aminocaprylic acid (0.86 g, 3.32 mmol) was added to a solution of 1,1'-carbonyldiimidazole (0.595 g, 3.67 mmol) in chloroform (25 mL). The reaction solution was stirred for 2 h at room temperature under nitrogen. A solution of *N*-Cbz-4,7,10-trioxa-1,13-tridecanediamine (1.30 g, 3.67 mmol) in chloroform (25 mL) was added to the solution and then stirred at room temperature for 16 h. The solution was evaporated under vacuum. The residue was dissolved in dichloromethane, washed with brine, dried over $MgSO_4$, filtered, and evaporated under vacuum. The crude product was purified by silica gel chromatography (from EA:Hex = 3:1 to DCM:MeOH = 10:1) to give **1** (1.6 g, 81%) as a clear oil. ^{1}H NMR (400 MHz, $CDCl_3$) δ 7.33-7.28 (m, 5H, Cbz), 5.07 (s, 2H, Cbz), 3.62-3.42 (m, 12H, C**H₂**OC**H₂**C**H₂**OC**H₂**C**H₂**OC**H₂**), 3.30 (m, 4H, NHC**H₂**CH_2CH_2O, CH_2C**H₂**NHBoc), 3.06 (br m, 2H, OCH_2CH_2C**H₂**NHCO), 2.13 (t, J = 7.6, 2H, NHCOC**H₂**), 1.80-1.70 (m, 4H, OCH_2C**H₂**CH_2NH), 1.59 (br m, 2H, $COCH_2$C**H₂**), 1.43 (br s, 11H, C(C**H₃**)₃, C**H₂**CH_2NHBoc), 1.28 (br s, 6H, C**H₂**C**H₂**C**H₂**CH_2CH_2NHBoc); ^{13}C NMR (100 MHz, $CDCl_3$) δ 173.2 (NH**C**OCH_2), 156.6 (O**C**ONH), 156.1 (O**C**ONH), 136.8 (Cbz), 128.4 (Cbz), 128.2 (Cbz), 127.9 (Cbz), 78.7 (**C**(CH_3)₃), 70.4 (two lines, O**C**H₂**C**H₂O), 70.1 (O**C**H₂CH₂O), 70.0 (O**C**H₂**C**H₂O), 69.7 (O**C**H₂CH₂CH₂), 69.4 (O**C**H₂CH₂CH₂), 66.3 (Cbz), 40.5 (NH**C**H₂), 39.0 (NH**C**H₂), 37.5 (NH**C**H₂), 36.5 (CO**C**H₂), 29.9 (COCH₂CH₂CH₂**C**H₂), 29.5 (COCH₂CH₂**C**H₂), 29.1 (two lines, OCH₂**C**H₂CH₂), 29.0, (**C**H₂CH₂CH₂NHBoc), 28.4 (C(**C**H₃)₃), 26.6 (**C**H₂CH₂NHBoc), 25.6 (COCH₂**C**H₂); MS (ESI-TOF) calcd for $C_{31}H_{53}N_3O_8$ 595.3833, found 596.1948 $(M + H)^+$.

Compound 2. A solution of **1** (1.30 g, 2.18 mmol) in dichloromethane (15 mL) and trifluoroacetic acid (15 mL) was stirred for 2 h at room temperature and then evaporated under vacuum. The residue was dissolved in chloroform, washed with 1 M NaOH (aq), dried over

MgSO4, filtered, and evaporated under vacuum to give the deprotected amine (1.08 g, quantitative) as a white solid. DUPA-tris (t-Butyl ester) (0.550 g, 1.13 mmol), the deprotected amine (0.558 g, 1.13 mmol), and HBTU (0.850 g, 2.24 mmol) were suspended in dichloromethane (20 mL) under argon. DIPEA (0.78 mL, 4.49 mmol) was slowly added to the suspension in an ice bath and stirred for 30 min. The suspension was stirred at room temperature for an additional 16 h. The solution was evaporated under vacuum. The residue was dissolved in ethyl acetate, washed with 0.5 M HCl (aq), dried over $MgSO_4$, filtered, and evaporated under vacuum. The crude product was purified by silica gel chromatography (DCM:MeOH = 10:1) to give **2** (1.0 g, 92%) as a clear oil. ^{1}H NMR (400 MHz, $CDCl_3$) δ 7.35-7.29 (m, 5H, Cbz), 5.09 (s, 2H, Cbz), 4.36-4.27 (m, 2H, $COC\mathbf{H}CH_2CH_2CO$ of DUPA), 3.63-3.49 (m, 12H, $C\mathbf{H_2}OC\mathbf{H_2}C\mathbf{H_2}OC\mathbf{H_2}C\mathbf{H_2}OC\mathbf{H_2}$), 3.32 (m, 4H, $CbzNHC\mathbf{H_2}CH_2CH_2O$, $CH_2C\mathbf{H_2}NH$-DUPA), 3.19 (m, 2H, $OCH_2CH_2C\mathbf{H_2}NHCO$), 2.32 (m, 2H, $COCHCH_2C\mathbf{H_2}COO$ of DUPA), 2.22-2.06 (m, 6H, $NHCOC\mathbf{H_2}$, $COCHC\mathbf{H_2}CH_2COO$ and $COCHC\mathbf{H_2}CH_2CONH$ of DUPA), 1.88-1.71 (m, 6H, $OCH_2C\mathbf{H_2}CH_2NH$, $COCHCH_2C\mathbf{H_2}CONH$ of DUPA), 1.60 (br m, 2H, $COCH_2C\mathbf{H_2}CH_2$), 1.46-1.43 (br m, 29H, $C(C\mathbf{H_3})_3$, $CH_2CH_2C\mathbf{H_2}CH_2NH$), 1.30 (br s, 6H, $C\mathbf{H_2}C\mathbf{H_2}C\mathbf{H_2}CH_2CH_2NH$-DUPA); ^{13}C NMR (100 MHz, $CDCl_3$) δ 173.2 ($NH\mathbf{C}OCH_2$), 172.8 ($CH_2\mathbf{C}ONH$ of DUPA), 172.4 (**C**OO of DUPA), 172.2 (**C**OO of DUPA), 171.9 (**C**OO of DUPA), 157.8 (NH**C**ONH of DUPA), 156.5 (O**C**ONH), 136.7 (Cbz), 128.4 (Cbz), 128.0 (two lines, Cbz), 82.1 ($\mathbf{C}(CH_3)_3$), 81.8 ($\mathbf{C}(CH_3)_3$), 80.5 ($\mathbf{C}(CH_3)_3$), 70.5 ($O\mathbf{C}H_2\mathbf{C}H_2O$), 70.4 ($O\mathbf{C}H_2\mathbf{C}H_2O$), 70.1 (two lines, $O\mathbf{C}H_2\mathbf{C}H_2O$), 69.9 ($O\mathbf{C}H_2CH_2CH_2$), 69.6 ($O\mathbf{C}H_2CH_2CH_2$), 66.4 (Cbz), 53.0 ($CO\mathbf{C}HCH_2CH_2CO$ of DUPA), 52.9 ($CO\mathbf{C}HCH_2CH_2CO$ of DUPA), 39.4 ($NH\mathbf{C}H_2$), 39.2 ($NH\mathbf{C}H_2$), 37.7 ($NH\mathbf{C}H_2$), 36.6 ($CO\mathbf{C}H_2CH_2$CH2), 32.5 ($COCHCH_2\mathbf{C}H_2CONH$ of DUPA), 31.6 ($COCHCH_2\mathbf{C}H_2COO$ of DUPA), 29.9 ($COCH_2CH_2CH_2\mathbf{C}H_2$), 29.4 (two lines, $OCH_2\mathbf{C}H_2CH_2$), 29.1 ($COCH_2CH_2\mathbf{C}H_2$), 29.0

(COCH**C**$H_2$$CH_2$CONH of DUPA), 28.8 (COCH**C**$H_2$$CH_2$COO of DUPA), 28.0 (three lines, (C(**C**H_3)$_3$), 26.6 (**C**$H_2$$CH_2$NH-DUPA), 25.6 (COC$H_2$**C**$H_2$$CH_2$); MS (ESI-TOF) calcd for $C_{49}H_{83}N_5O_{14}$ 965.5937, found 966.3426 $(M + H)^+$.

Compound 3. Compound **2** (0.70 g, 0.724 mmol) was dissolved in ethyl acetate (10 mL) and methanol (10 mL) and followed by addition of 10% Pd on activated charcoal (70 mg). The reaction vessel was repeatedly degassed and flushed with hydrogen gas. The reaction mixture was stirred at room temperature with a hydrogen balloon for 24 h, filtered through a celite layer, and evaporated under vacuum. The resulting oil was triturated with hexane to give **3** (0.60 g, quantitative) as a white semi-solid. ^{1}H NMR (400 MHz, $CDCl_3$) δ 4.31-4.24 (br m, 2H, CO**C****H**$CH_2$$CH_2$CO of DUPA), 3.63-3.49 (m, 12H, C**H_2**OC**H_2**C**H_2**OC**H_2**C**H_2**OC**H_2**), 3.32 (m, 2H, , CH_2C**H_2**NH-DUPA), 3.20 (m, 4H, NH_2C**H_2**$CH_2$$CH_2O, OCH_2$$CH_2$C**$H_2$**NHCO), 2.36-2.31 (m, 4H, OCH_2C**H_2**$CH_2$$NH_2$, COCH$CH_2$C**$H_2$**COO of DUPA), 2.23 (t, J = 7.4, 2H, NHCOC**H_2**$CH_2$$CH_2$, 2.12-2.03 (m, 4H, COCHC**H_2**CH_2COO and COCHC**H_2**CH_2CONH of DUPA), 1.90-1.78 (m, 4H, OCH_2C**H_2**CH_2NHCO, COCHCH_2C**H_2**CONH of DUPA), 1.61 (br m, 2H, COCH_2C**H_2**CH_2), 1.50-1.43 (br m, 29H, C(C**H_3**)$_3$, $CH_2$$CH_2$C**$H_2$**$CH_2$NH), 1.32 (br s, 6H, C**$H_2$**C**$H_2$**C**$H_2$**$CH_2$$CH_2$NH-DUPA); ^{13}C NMR (100 MHz, $CDCl_3$) δ 174.2 (NH**C**OCH_2), 173.0 (CH_2**C**ONH of DUPA), 172.6 (**C**OO of DUPA), 172.3 (**C**OO of DUPA), 172.1 (**C**OO of DUPA), 157.9 (NH**C**ONH of DUPA), 81.8 (**C**(CH_3)$_3$), 81.6 (**C**(CH_3)$_3$), 80.4 (**C**(CH_3)$_3$), 70.3 (O**C**H_2**C**H_2O), 69.9 (O**C**H_2**C**H_2O), 69.7 (O**C**H_2**C**H_2O), 69.6 (O**C**$H_2$$CH_2$$CH_2$), 69.0 (O**C**$H_2$$CH_2$$CH_2$), 53.2 (CO**C**H$CH_2$$CH_2$CO of DUPA), 53.0 (CO**C**H$CH_2$$CH_2$CO of DUPA), 39.7 (NH**C**H_2), 39.4 (NH**C**H_2), 36.8 (CO**C**$H_2$$CH_2$CH2), 36.2 (**C**$H_2$$NH_2$), 32.5 (COCH$CH_2$**C**$H_2$CONH of DUPA), 31.6 (COCHCH_2**C**H_2COO of DUPA), 29.5 (COC$H_2$$CH_2$$CH_2$**C**$H_2$), 29.2 (O$CH_2$**C**$H_2$$CH_2$), 29.0 (COC$H_2$$CH_2$**C**$H_2$), 28.7 (COCH**C**$H_2$$CH_2$CONH of DUPA), 28.4 (COCH**C**$H_2$$CH_2$COO of DUPA), 28.0 (three lines,

(C(**C**H_3)$_3$), 26.4 (**C**$H_2$$CH_2$NH-DUPA), 25.4 (COC$H_2$**C**$H_2$$CH_2$); MS (ESI-TOF) calcd for $C_{41}H_{77}N_5O_{12}$ 831.5569, found 832.5791 $(M + H)^+$.

DUPA-DCT. Cyanuric chloride (0.122 g, 0.662 mmol) was added to a solution of **3** (0.500 g, 0.600 mmol) and DIPEA (0.23 mL, 1.31 mmol) in THF (20 mL) at 0 ^oC, The solution was stirred at 0 ^oC for 1 h and evaporated under vacuum. The residue was dissolved in dichloromethane, washed with brine, dried over $MgSO_4$, filtered, and evaporated under vacuum. The crude product was purified by silica gel chromatography (DCM:MeOH = 12:1) to give **DUPA-DCT** (0.53 g, 90%) as a white sticky solid. ^{1}H NMR (400 MHz, $CDCl_3$) δ 4.33-4.23 (br m, 2H, CO**CH**$CH_2$$CH_2$CO of DUPA), 3.70-3.56 (m, 14H, C_3N_3-NHC**H_2**$CH_2$$CH_2$O, C**$H_2$**OC**$H_2$**C**$H_2$**OC**$H_2$**C**$H_2$**OC**$H_2$**), 3.37 (m, 2H, CH_2C**H_2**NH-DUPA), 3.25 (m, 2H, O$CH_2$$CH_2$C**$H_2$**NHCO), 2.41-2.05 (m, 8H, COCHCH_2C**H_2**COO of DUPA, NHCOC**H_2**$CH_2$$CH_2$, COCHC**$H_2$**$CH_2$COO and COCHC**$H_2$**$CH_2$CONH of DUPA), 1.93-1.78 (m, 6H, OCH_2C**H_2**CH_2NH-C_3N_3, OCH_2C**H_2**CH_2NHCO, COCHCH_2C**H_2**CONH of DUPA), 1.63 (br, 2H, COCH_2C**H_2**CH_2), 1.54-1.44 (br m, 29H, C(C**H_3**)$_3$, $CH_2$$CH_2$C**$H_2$**$CH_2$NH), 1.32 (br s, 6H, C**$H_2$**C**$H_2$**C**$H_2$**$CH_2$$CH_2$NH-DUPA); ^{13}C NMR (100 MHz, $CDCl_3$) δ 174.0 (NH**C**OCH_2), 173.6 (CH_2**C**ONH of DUPA), 172.1 (**C**OO of DUPA), 171.2 (**C**OO of DUPA), 170.5 (**C**OO of DUPA), 169.5 (C_3N_3), 165.5 (C_3N_3), 158.3 (NH**C**ONH of DUPA), 82.1 (**C**(CH_3)$_3$), 82.0 (**C**(CH_3)$_3$), 80.5 (**C**(CH_3)$_3$), 70.5 (O**C**H_2**C**H_2O), 70.42 (O**C**H_2**C**H_2O), 70.2 (O**C**H_2**C**H_2O), 70.1 (O**C**H_2**C**H_2O), 69.7 (O**C**$H_2$$CH_2$$CH_2$), 69.6 (O**C**$H_2$$CH_2$$CH_2$), 53.2 (CO**C**H$CH_2$$CH_2$CO of DUPA), 52.9 (CO**C**H$CH_2$$CH_2$CO of DUPA), 40.0 (NH**C**H_2), 39.9 (NH**C**H_2), 37.9 (NH**C**H_2), 36.1 (CO**C**$H_2$$CH_2$$CH_2$), 31.5 (COCH$CH_2$**C**$H_2$CONH of DUPA), 30.0 (COCHCH_2**C**H_2COO of DUPA), 28.9 (br two lines, CO$CH_2$$CH_2$$CH_2$**C**$H_2$, O$CH_2$**C**$H_2$$CH_2$, CO$CH_2$$CH_2$**C**$H_2$), 28.5 (COCH**C**$H_2$$CH_2$CONH of DUPA), 28.3 (COCH**C**$H_2$$CH_2$COO of DUPA), 28.0 (two lines, (C(**C**H_3)$_3$), 27.9 (C(**C**H_3)$_3$), 26.4 (**C**$H_2$$CH_2$NH-

DUPA), 25.6 ($COCH_2\mathbf{C}H_2CH_2$); MS (ESI-TOF) calcd for $C_{44}H_{76}Cl_2N_8O_{12}$ 978.4960, found 979.6901 $(M + H)^+$.

G1 Platform. DOTAGA-tetra (t-Bu ester) (0.189 g, 0.270 mmol), **Triazine Core** (0.440 g, 0.270 mmol), and HBTU (0.204 g, 0.538 mmol) were suspended in dichloromethane (20 mL) under argon. DIPEA (0.20 mL, 1.15 mmol) was slowly added to the suspension in an ice bath and stirred for 30 min. The suspension was stirred at room temperature for an additional 16 h. The solution was evaporated under vacuum. The residue was dissolved in ethyl acetate, washed with 0.1 M HCl (aq), dried over $MgSO_4$, filtered, and evaporated under vacuum. The crude product was purified by silica gel chromatography (EA:DCM:MeOH = 4.5:4.5:1) to give **G1 Platform** (0.53 g, 85%) as a white solid. ^1H NMR (400 MHz, $CDCl_3$) δ 3.73-3.26 (br m, 87H, $NC\mathbf{H_2}CH_2NBoc$, $C\mathbf{H_2}OC\mathbf{H_2}C\mathbf{H_2}OC\mathbf{H_2}C\mathbf{H_2}OC\mathbf{H_2}$, $NHC\mathbf{H_2}CH_2CH_2O$, $NCH_2C\mathbf{H_2}NBoc$, $C\mathbf{H}COO$ and $C\mathbf{H_2}COO$ of DOTA), 3.01-2.10 (m, 20H, DOTA), 1.83 (m, 12H, $OCH_2C\mathbf{H_2}CH_2NH$), 1.47 (s, 72H, $C(C\mathbf{H_3})_3$); ^{13}C NMR (100 MHz, $CDCl_3$) δ 176.6 (NH**C**O of DOTA), 175.1 (**C**OO of DOTA), 172.8 (two lines, (**C**OO of DOTA), 172.6 (**C**OO of DOTA), 165.8 ($\mathbf{C}_3N_3$), 165.0 ($\mathbf{C}_3N_3$), 154.8 (N**C**OO), 82.3 ($\mathbf{C}(CH_3)_3$ of DOTA), 82.0 ($\mathbf{C}(CH_3)_3$ of DOTA), 81.9 (two lines, $\mathbf{C}(CH_3)_3$ of DOTA), 79.8 ($\mathbf{C}(CH_3)_3$ of Boc), 70.4 ($O\mathbf{C}H_2CH_2O$), 70.1 ($O\mathbf{C}H_2CH_2O$), 69.9 ($O\mathbf{C}H_2CH_2O$), 69.3 ($NHCH_2CH_2\mathbf{C}H_2O$), 69.1 ($NHCH_2CH_2\mathbf{C}H_2O$), 60.3 (**C**H of DOTA), 55.8 (DOTA), 55.7 (DOTA), 55.5 (DOTA), 52.6 (DOTA), 52.5 (DOTA), 48.3 (DOTA), 47.9 (DOTA), 47.1 (DOTA), 44.1 (br, piperazine), 42.9 (piperazine), 38.2 (br m, $NH\mathbf{C}H_2$), 37.0 (DOTA), 34.6 (DOTA), 29.5 ($NHCH_2\mathbf{C}H_2CH_2O$), 28.9 (($NHCH_2\mathbf{C}H_2CH_2O$), 28.4 ($C(\mathbf{C}H_3)_3$), 27.9 ($C(\mathbf{C}H_3)_3$), 27.8 ($C(\mathbf{C}H_3)_3$), 27.7 ($C(\mathbf{C}H_3)_3$), 22.2 (DOTA); MS (ESI-TOF) calcd for $C_{110}H_{197}N_{27}O_{26}$ 2312.4923, found 2313.5491 $(M + H)^+$.

G3 Platform. A solution of **G1 Platform** (0.17 g, 73.50 μmol) in 4 M HCl in dioxane (4 mL) was stirred at room temperature for 2 h and then evaporated under vacuum. The residue was dissolved in chloroform, washed with 1 M NaOH (aq), dried over $MgSO_4$, filtered, and evaporated under vacuum. The resulting compound was dissolved in a solution of **Macromonomeric MCT** (0.595 g, 0.411 mmol) and DIPEA (0.14 mL, 0.804 mmol) in THF (3 mL), methanol (0.15 mL), and H_2O (0.15 mL) and refluxed for 48 h. The solution was evaporated under vacuum. The residue was dissolved in dichloromethane, washed with brine, dried over $MgSO_4$, filtered, and evaporated under vacuum. The crude product was purified by silica gel chromatography (from Ace:Hex = 2:1 to DCM:MeOH = 7:1) to give **G3 Platform** (0.46 g, 83 % over two steps) as a white solid. ^{1}H NMR (400 MHz, $CDCl_3$) δ 3.73-3.26 (br m, 343H, C_3N_3-NC**H$_2$**C**H$_2$**N, NC**H$_2$**CH_2NBoc, C**H$_2$**OC**H$_2$**C**H$_2$**OC**H$_2$**C**H$_2$**OC**H$_2$**, NHC**H$_2$**CH_2CH_2O, NCH_2C**H$_2$**NBoc, C**H**COO and C**H$_2$**COO of DOTA), 3.01-2.10 (m, 20H, DOTA with weak signals), 1.83 (br m, 44H, OCH_2C**H$_2$**CH_2NH), 1.48 (br m, 180H, C(C**H$_3$**)$_3$); ^{13}C NMR (100 MHz, $CDCl_3$) δ 175.7 (DOTA with a weak signal), 173.0 (DOTA with a weak signal), 172.8 (DOTA with a weak signal), 172.7 (DOTA with a weak signal), 172.5 (DOTA with a weak signal), 166.3 (**C**$_3N_3$), 165.2 (**C**$_3N_3$), 154.8 (N**C**OO), 82.1 (**C**$(CH_3)_3$ of DOTA with a weak signal), 82.0 (two lines, **C**$(CH_3)_3$ of DOTA with a weak signal), 81.9 (**C**$(CH_3)_3$ of DOTA with a weak signal), 79.8 (**C**$(CH_3)_3$ of Boc), 70.6 (O**C**H_2**C**H_2O), 70.2 (br two lines, O**C**H_2**C**H_2O), 69.3 (br two lines, $NHCH_2CH_2$**C**H_2O), 60.5 (**C**H of DOTA with a weak signal), 55.8 (DOTA with a weak signal), 55.5 (DOTA with a weak signal), 52.7. (DOTA with a weak signal), 47.0 (DOTA with a weak signal), 44.0 (br, piperazine), 42.9 (piperazine), 38.2 (NH**C**H_2), 38.1 (NH**C**H_2), 36.8 (DOTA with a weak signal), 29.6 ($NHCH_2$**C**H_2CH_2O), 28.4 (C(**C**H_3)$_3$ of Boc), 27.9 (two lines, C(**C**H_3)$_3$ of DOTA), 27.8 (two lines, C(**C**H_3)$_3$ of DOTA); MS (ESI-TOF) calcd for $C_{350}H_{609}N_{111}O_{74}$ 7551.7303, found 7553.5735 $(M + H)^+$.

G5 Platform. A solution of **G3 Platform** (70 mg, 9.26 μmol) in 4 M HCl in dioxane (4 mL) was stirred at room temperature for 4 h and then evaporated under vacuum. The residue was dissolved in chloroform, washed with 1 M NaOH (aq), dried over $MgSO_4$, filtered, and evaporated under vacuum. The resulting compound was dissolved in a solution of **Macromonomeric MCT** (0.30 g, 0.207 mmol) and DIPEA (0.14 mL, 0.804 mmol) in THF (3 mL), methanol (0.15 mL), and H_2O (0.15 mL) and refluxed for 48 h. The solution was evaporated under vacuum. The residue was dissolved in dichloromethane, washed with brine, dried over $MgSO_4$, filtered, and evaporated under vacuum. The crude product was purified by silica gel chromatography (from Ace:Hex = 2:1 to DCM:MeOH = 7:1) to give **G5 Platform** (0.20 g, 76% over two steps) as a white solid. ^1H NMR (400 MHz, $CDCl_3$) δ 3.73-3.43 (br m, 1360H, C_3N_3-NC$\mathbf{H_2}$C$\mathbf{H_2}$N, NC$\mathbf{H_2}$$CH_2$NBoc, C$\mathbf{H_2}OC\mathbf{H_2}C\mathbf{H_2}OC\mathbf{H_2}C\mathbf{H_2}OC\mathbf{H_2}$, NHC$\mathbf{H_2}$$CH_2CH_2O$, N$CH_2C\mathbf{H_2}$NBoc), 1.83 (br m, 172H, OCH_2C$\mathbf{H_2}$$CH_2$NH), 1.48 (612H, C(C$\mathbf{H_3}$)$_3$); ^{13}C NMR (100 MHz, $CDCl_3$) δ 166.3 (**C**$_3N_3$), 165.2 (**C**$_3N_3$), 154.8 (N**C**OO), 79.8 (**C**$(CH_3)_3$ of Boc), 70.6 (O**C**H_2**C**H_2O), 70.2 (br, O**C**H_2**C**H_2O), 69.3 (br two lines, NHCH_2CH_2**C**H_2O), 44.0 (br, piperazine), 42.9 (piperazine), 38.2 (NH**C**H_2), 38.1 (NH**C**H_2), 29.6 (NHCH_2**C**H_2CH_2O), 28.4 (C(**C**H_3)$_3$ of Boc), 27.9 (br, C(**C**H_3)$_3$ of DOTA with a weak signal); MS (ESI-TOF) calcd for $C_{1310}H_{2257}N_{447}O_{266}$ 28508.68, found 28521.13 $(M + H)^+$.

Compound 4. A solution of **G1 Platform** (0.13 g, 56.2 μmol) in 4 M HCl in dioxane (4 mL) was stirred at room temperature for 2 h and then evaporated under vacuum. The residue was dissolved in chloroform, washed with 1 M NaOH (aq), dried over MgSO4, filtered, and evaporated under vacuum. A solution of the deprotected G1 platform and DIPEA (0.14 mL, 0.804 mmol) in THF (3 mL), methanol (0.2 mL), and H_2O (0.2 mL) was cooled in an ice bath before **DUPA-DCT** (0.33 g, 0.337 mmol) was added. The reaction solution was warmed to room temperature and stirred for 24 h. The solution was evaporated under vacuum. The residue was dissolved in

dichloromethane, washed with brine, dried over $MgSO_4$, filtered, and evaporated under vacuum. The crude product was purified by silica gel chromatography (from Ace:Hex = 2:1 to DCM:MeOH = 7:1) to give **4** (0.27 g, 84% over two steps) as a white solid. ^{1}H NMR (400 MHz, $CDCl_3$) δ 4.34-4.23 (br m, 8H, COC**H**CH_2CH_2CO of DUPA), 3.84-3.19 (m, 159H, C_3N_3-NHC$\mathbf{H_2}$$CH_2CH_2O, NC\mathbf{H_2}C\mathbf{H_2}N, C\mathbf{H_2}OC\mathbf{H_2}C\mathbf{H_2}OC\mathbf{H_2}C\mathbf{H_2}OC\mathbf{H_2}$, CH_2C$\mathbf{H_2}$NH, C**H**COO and C$\mathbf{H_2}$COO of DOTA), 3.11-2.05 (m, 52H, COCHCH_2C$\mathbf{H_2}$COO of DUPA, NHCOC$\mathbf{H_2}$$CH_2CH_2$, COCHC$\mathbf{H_2}$$CH_2$COO and COCHC$\mathbf{H_2}$$CH_2$CONH of DUPA, DOTA), 1.90-1.76 (m, 36H, OCH_2C$\mathbf{H_2}$$CH_2$NH, COCH$CH_2C\mathbf{H_2}$CONH of DUPA), 1.61 (br, 8H, COCH_2C$\mathbf{H_2}$$CH_2$), 1.52-1.43 (br m, 152H, C(C$\mathbf{H_3}$)$_3$, CH_2CH_2C$\mathbf{H_2}$$CH_2$NH), 1.31 (br s, 24H, C$\mathbf{H_2}C\mathbf{H_2}C\mathbf{H_2}$$CH_2CH_2$NH-DUPA); ^{13}C NMR (100 MHz, $CDCl_3$) δ 175.2 (**C**OO of DOTA with a weak signal), 173.5 (NH**C**OCH_2), 173.3 (NH**C**OCH_2), 172.7 (br, **C**OO of DOTA), 172.6 (**C**OO of DUPA), 172.3 (**C**OO of DUPA), 172.0 (**C**OO of DUPA), 168.9 (C_3N_3), 166.2 (C_3N_3), 165.4 ($\mathbf{C}_3N_3$), 165.2 ($\mathbf{C}_3N_3$), 164.5 ($\mathbf{C}_3N_3$), 157.9 (NH**C**ONH of DUPA), 82.2 (**C**(CH_3)$_3$ of DOTA), 82.1 (**C**(CH_3)$_3$ of DOTA), 82.0 (two lines, **C**(CH_3)$_3$ of DOTA), 81.8 (**C**(CH_3)$_3$ of DUPA), 81.5 (**C**(CH_3)$_3$ of DUPA), 80.4 (**C**(CH_3)$_3$ of DUPA), 70.5 (O**C**H_2CH_2O), 70.4 (O**C**H_2**C**H_2O), 70.1 (O**C**H_2**C**H_2O), 70.0 (O**C**H_2**C**H_2O), 69.8 (O**C**H_2CH_2O), 69.4 (NHCH_2CH_2**C**H_2O), 69.3 (NHCH_2CH_2**C**H_2O), 69.2 (NHCH_2CH_2**C**H_2O), 60.0 (**C**H of DOTA with a weak signal), 55.7 (DOTA with a weak signal), 55.4 (DOTA with a weak signal), 53.5 (DOTA with a weak signal), 53.0 (CO**C**HCH_2CH_2CO of DUPA), 52.7 (DOTA with a weak signal), 48.4 (DOTA with a weak signal), 47.1 (DOTA with a weak siganl), 43.3 (br, piperazine), 42.8 (br, piperazine), 41.9 (NH**C**H_2), 39.3 (NH**C**H_2), 38.9 (NH**C**H_2), 38.2 (NH**C**H_2), 38.1 (NH**C**H_2), 37.5 (NH**C**H_2), 37.3 (DOTA with a weak signal), 36.6 (CO**C**$H_2CH_2CH_2$), 32.6 (COCHCH_2**C**H_2CONH of DUPA), 31.6 (COCHCH_2**C**H_2COO of DUPA), 29.7 (NHCH_2**C**H_2CH_2O), 29.5 (NHCH_2**C**H_2CH_2O), 29.3 (NHCH_2**C**H_2CH_2O), 29.0 (COCH_2CH_2**C**H_2,

COCH**C**$H_2$$CH_2$CONH of DUPA), 28.7 (COC$H_2CH_2CH_2$**C**$H_2$, COCH**C**$H_2CH_2$CONH of DUPA), 28.0 (two lines, C(**C**H_3)$_3$), 27.9 (C(**C**H_3)$_3$), 27.8 (two lines, C(**C**H_3)$_3$), 26.6 (**C**H_2CH_2NH-DUPA), 25.6 (COCH_2**C**H_2CH_2); MS (ESI-TOF) calcd for $C_{266}H_{465}Cl_4N_{59}O_{66}$ 5682.3598, found 5687.7884 $(M + H)^+$.

Compound 5. A solution of **G3 Platform** (0.13 g, 17.2 μmol) in 4 M HCl in dioxane (4 mL) was stirred at room temperature for 4 h and then evaporated under vacuum. The residue was dissolved in chloroform, washed with 1 M NaOH (aq), dried over MgSO4, filtered, and evaporated under vacuum. A solution of the deprotected G3 platform and DIPEA (0.19 mL, 1.09 mmol) in THF (3 mL), methanol (0.2 mL), and H_2O (0.2 mL) was cooled in an ice bath before **DUPA-DCT** (0.405 g, 0.413 mmol) was added. The reaction solution was warmed to room temperature and stirred for 48 h. The solution was evaporated under vacuum. The residue was dissolved in dichloromethane, washed with brine, dried over $MgSO_4$, filtered, and evaporated under vacuum. The crude product was purified by silica gel chromatography (from Ace:Hex = 2:1 to DCM:MeOH = 7:1) to give **5** (0.28 g, 77% over two steps) as a white solid. ^{1}H NMR (400 MHz, $CDCl_3$) δ 4.36-4.27 (br m, 32H, COC**H**CH_2CH_2CO of DUPA), 3.84-3.19 (m, 631H, C_3N_3-NHC**H₂**CH_2CH_2O, NC**H₂**C**H₂**N, C**H₂**OC**H₂**C**H₂**OC**H₂**C**H₂**OC**H₂**, CH_2C**H₂**NH, C**H**COO and C**H₂**COO of DOTA), 3.11-2.05 (m, 148H, COCHCH_2C**H₂**COO of DUPA, NHCOC**H₂**CH_2CH_2, COCHC**H₂**CH_2COO and COCHC**H₂**CH_2CONH of DUPA, DOTA), 1.90-1.76 (m, 140H, OCH_2C**H₂**CH_2NH, COCHCH_2C**H₂**CONH of DUPA), 1.60 (br, 32H, COCH_2C**H₂**CH_2), 1.52-1.43 (br m, 464H, C(C**H₃**)$_3$, CH_2CH_2C**H₂**CH_2NH), 1.31 (br s, 96H, C**H₂**C**H₂**C**H₂**CH_2CH_2NH-DUPA); ^{13}C NMR (100 MHz, $CDCl_3$) δ 173.2 (NH**C**OCH_2), 172.8 (NH**C**OCH_2), 172.4 (**C**OO of DUPA), 172.2 (**C**OO of DUPA), 172.0 (**C**OO of DUPA), 168.9 (C_3N_3), 166.2 (C_3N_3), 165.4 (**C**$_3N_3$), 165.2 (**C**$_3N_3$), 164.5 (**C**$_3N_3$), 157.9 (NH**C**ONH of DUPA), 82.0 (**C**(CH_3)$_3$ of DUPA), 81.6 (**C**(CH_3)$_3$ of DUPA),

80.5 ($\mathbf{C}(CH_3)_3$ of DUPA), 70.5 ($O\mathbf{C}H_2\mathbf{C}H_2O$), 70.4 ($O\mathbf{C}H_2\mathbf{C}H_2O$), 70.2 ($O\mathbf{C}H_2\mathbf{C}H_2O$), 70.1 ($O\mathbf{C}H_2\mathbf{C}H_2O$), 69.8 ($O\mathbf{C}H_2\mathbf{C}H_2O$), 69.4 ($NHCH_2CH_2\mathbf{C}H_2O$), 69.3 ($NHCH_2CH_2\mathbf{C}H_2O$), 53.0 ($CO\mathbf{C}HCH_2CH_2CO$ of DUPA), 52.9 ($CO\mathbf{C}HCH_2CH_2CO$ of DUPA), 43.3 (br, piperazine), 42.8 (br, piperazine), 41.7 ($NH\mathbf{C}H_2$), 39.3 ($NH\mathbf{C}H_2$), 38.8 ($NH\mathbf{C}H_2$), 38.2 ($NH\mathbf{C}H_2$), 38.1 ($NH\mathbf{C}H_2$), 37.6 ($NH\mathbf{C}H_2$), 36.6 ($CO\mathbf{C}H_2CH_2CH_2$), 32.5 ($COCHCH_2\mathbf{C}H_2CONH$ of DUPA), 31.6 ($COCHCH_2\mathbf{C}H_2COO$ of DUPA), 29.8 ($NHCH_2\mathbf{C}H_2CH_2O$), 29.5 ($NHCH_2\mathbf{C}H_2CH_2O$), 29.3 ($NHCH_2\mathbf{C}H_2CH_2O$), 29.1 ($COCH_2CH_2\mathbf{C}H_2$), 29.0 ($COCH\mathbf{C}H_2CH_2CONH_2$ of DUPA), 28.8 ($COCH_2CH_2CH_2\mathbf{C}H_2$), 28.7 ($COCH\mathbf{C}H_2CH_2CONH$ of DUPA), 28.0 (two lines, $C(\mathbf{C}H_3)_3$), 27.9 ($C(\mathbf{C}H_3)_3$), 27.8 ($C(\mathbf{C}H_3)_3$), 27.7 ($C(\mathbf{C}H_3)_3$), 26.6 ($\mathbf{C}H_2CH_2NH$-DUPA), 25.6 ($COCH_2\mathbf{C}H_2CH_2$); MS (ESI-TOF) calcd for $C_{974}H_{1681}C_{116}N_{239}O_{234}$ 21031.20, found 21055.31 $(M + H)^+$.

Compound 6. A solution of **G5 Platform** (0.15 g, 5.26 μmol) in 4 M HCl in dioxane (4 mL) was stirred at room temperature for 8 h and then evaporated under vacuum. The residue was dissolved in chloroform, washed with 1 M NaOH (aq), dried over MgSO4, filtered, and evaporated under vacuum. A solution of the deprotected G5 platform and DIPEA (0.19 mL, 1.09 mmol) in THF (3 mL), methanol (0.2 mL), and H_2O (0.2 mL) was cooled in an ice bath before **DUPA-DCT** (0.495 g, 0.505 mmol) was added. The reaction solution was warmed to room temperature and stirred for 48 h. The solution was evaporated under vacuum. The residue was dissolved in dichloromethane, washed with brine, dried over $MgSO_4$, filtered, and evaporated under vacuum. The crude product was purified by silica gel chromatography (from Ace:Hex = 2:1 to DCM:MeOH = 7:1) to give **6** (0.30 g, 69% over two steps) as a white solid. ^1H NMR (400 MHz, $CDCl_3$) δ 4.34-4.27 (br m, 128H, $COC\mathbf{H}CH_2CH_2CO$ of DUPA), 3.84-3.16 (m, 2512H, C_3N_3-$NHC\mathbf{H_2}CH_2CH_2O$, $N\mathbf{CH_2CH_2}N$, $C\mathbf{H_2}OC\mathbf{H_2}C\mathbf{H_2}OC\mathbf{H_2}C\mathbf{H_2}OC\mathbf{H_2}$, $CH_2C\mathbf{H_2}NH$, $C\mathbf{H}COO$ and $C\mathbf{H_2}COO$ of DOTA), 3.11-2.06 (m, 532H, $COCHCH_2C\mathbf{H_2}COO$ of DUPA, $NHCOC\mathbf{H_2}CH_2CH_2$, $COCHC\mathbf{H_2}CH_2COO$

and $COCHCH_2CH_2CONH$ of DUPA, DOTA), 1.90-1.76 (m, 556H, $OCH_2C\mathbf{H_2}CH_2NH$, $COCHCH_2C\mathbf{H_2}CONH$ of DUPA), 1.59 (br, 128H, $COCH_2C\mathbf{H_2}CH_2$), 1.52-1.43 (br m, 1892H, $C(C\mathbf{H_3})_3$, $CH_2CH_2C\mathbf{H_2}CH_2NH$), 1.30 (br s, 384H, $C\mathbf{H_2}C\mathbf{H_2}C\mathbf{H_2}CH_2CH_2NH$-DUPA); ^{13}C NMR (100 MHz, $CDCl_3$) δ 173.3 ($NH\mathbf{C}OCH_2$), 172.8 ($NH\mathbf{C}OCH_2$), 172.4 (**C**OO of DUPA), 172.2 (**C**OO of DUPA), 172.0 (**C**OO of DUPA), 168.9 (C_3N_3), 166.3 (C_3N_3), 165.4 ($\mathbf{C}_3N_3$), 165.2 ($\mathbf{C}_3N_3$), 164.5 ($\mathbf{C}_3N_3$), 157.9 (NH**C**ONH of DUPA), 81.9 ($\mathbf{C}(CH_3)_3$ of DUPA), 81.7 ($\mathbf{C}(CH_3)_3$ of DUPA), 80.5 ($\mathbf{C}(CH_3)_3$ of DUPA), 70.5 ($O\mathbf{C}H_2\mathbf{C}H_2O$), 70.4 ($O\mathbf{C}H_2\mathbf{C}H_2O$), 70.2 ($O\mathbf{C}H_2\mathbf{C}H_2O$), 70.1 ($O\mathbf{C}H_2\mathbf{C}H_2O$), 69.9 ($O\mathbf{C}H_2\mathbf{C}H_2O$), 69.4 ($NHCH_2CH_2\mathbf{C}H_2O$), 69.3 ($NHCH_2CH_2\mathbf{C}H_2O$), 53.0 ($CO\mathbf{C}HCH_2CH_2CO$ of DUPA), 52.9 ($CO\mathbf{C}HCH_2CH_2CO$ of DUPA), 43.3 (br, piperazine), 42.9 (br, piperazine), 41.9 ($NH\mathbf{C}H_2$), 39.4 ($NH\mathbf{C}H_2$), 38.9 ($NH\mathbf{C}H_2$), 38.2 ($NH\mathbf{C}H_2$), 37.6 ($NH\mathbf{C}H_2$), 36.6 ($CO\mathbf{C}H_2CH_2CH_2$), 32.6 ($COCHCH_2\mathbf{C}H_2CONH$ of DUPA), 31.6 ($COCHCH_2\mathbf{C}H_2COO$ of DUPA), 29.8 ($NHCH_2\mathbf{C}H_2CH_2O$), 29.6 ($NHCH_2\mathbf{C}H_2CH_2O$), 29.4 ($NHCH_2\mathbf{C}H_2CH_2O$), 29.1 ($COCH_2CH_2\mathbf{C}H_2$), 29.0 ($COCH\mathbf{C}H_2CH_2CONH_2$ of DUPA), 28.8 (two lines, $COCH_2CH_2CH_2\mathbf{C}H_2$, $COCH\mathbf{C}H_2CH_2CONH$ of DUPA), 28.1 (two lines, $C(\mathbf{C}H_3)_3$), 28.0 ($C(\mathbf{C}H_3)_3$), 26.7 ($\mathbf{C}H_2CH_2NH$-DUPA), 25.6 ($COCH_2\mathbf{C}H_2CH_2$).

G1-(DUPA)$_4$. A solution of 4-(aminomethyl)piperidine (0.16 g, 1.40 mmol) in THF (2 mL) was added to a solution of **4** (0.167 g, 29.4 μmol) in THF (3 mL). The solution was stirred at room temperature for 16 h and then evaporated under vacuum. The residue was dissolved in dichloromethane (30 mL), washed three times with brine, dried over $MgSO_4$, filtered, and evaporated under vacuum. The resulting compound was dissolved in 3 mL of a mixture of TFA/TIPS/H_2O (95/2.5/2.5) and stirred at room temperature for 16 h. The solution was evaporated under vacuum. The residue was dissolved in methanol. The resulting solution was evaporated under vacuum to give **G1-(DUPA)$_4$** (0.15 g, quantitative) as a TFA salt. ^1H NMR (400 MHz,

CD_3OD) δ 4.34-4.23 (br m, 8H, $COC\mathbf{H}CH_2CH_2CO$ of DUPA), 4.01-3.14 (m, 167H, C_3N_3-$NHC\mathbf{H_2}CH_2CH_2O$, $NC\mathbf{H_2}C\mathbf{H_2}N$, $C\mathbf{H_2}OC\mathbf{H_2}C\mathbf{H_2}OC\mathbf{H_2}C\mathbf{H_2}OC\mathbf{H_2}$, $CH_2C\mathbf{H_2}NH$, $C\mathbf{H}COO$ and $C\mathbf{H_2}COO$ of DOTA, AMP), 3.04-2.05 (m, 68H, $COCHCH_2C\mathbf{H_2}COO$ of DUPA, $NHCOC\mathbf{H_2}CH_2CH_2$, $COCHC\mathbf{H_2}CH_2COO$ and $COCHC\mathbf{H_2}CH_2CONH$ of DUPA, DOTA, AMP), 1.91-1.74 (br m, 44H, $OCH_2C\mathbf{H_2}CH_2NH$, $COCHCH_2C\mathbf{H_2}CO$ of DUPA), 1.59 (br, 8H, $COCH_2C\mathbf{H_2}CH_2$), 1.49 (br, 8H, $CH_2CH_2C\mathbf{H_2}CH_2NH$), 1.39-1.26 (br m, 36H, $C\mathbf{H_2}C\mathbf{H_2}C\mathbf{H_2}CH_2CH_2NH$-DUPA, AMP); ^{13}C NMR (100 MHz, CD_3OD) δ 176.5 (two lines, **C**OO), 176.0 (two lines, **C**OO), 175.1 (br, $NH\mathbf{C}OCH_2$), 175.0 ($NH\mathbf{C}OCH_2$), 174.8 ($NH\mathbf{C}OCH_2$), 163.3 ($\mathbf{C}_3N_3$), 157.2 (NH**C**ONH of DUPA), 71.6 (two lines, $O\mathbf{C}H_2\mathbf{C}H_2O$), 71.3 (two lines, $O\mathbf{C}H_2\mathbf{C}H_2O$), 70.0 ($O\mathbf{C}H_2\mathbf{C}H_2O$), 69.9 ($O\mathbf{C}H_2\mathbf{C}H_2O$), 69.8 ($NHCH_2CH_2\mathbf{C}H_2O$), 69.7 ($NHCH_2CH_2\mathbf{C}H_2O$), 55.9 (DOTA with a weak signal), 54.1 (DOTA with a weak signal), 53.9 ($CO\mathbf{C}HCH_2CH_2CO$ of DUPA), 53.8 ($CO\mathbf{C}HCH_2CH_2CO$ of DUPA), 52.9 (DOTA), 52.4 (DOTA), 45.5 (br, AMP), 44.9 (br, piperazine), 43.8 (br, piperazine, AMP), 40.6 ($NH\mathbf{C}H_2$), 39.7 ($NH\mathbf{C}H_2$), 39.3 (AMP), 38.0 ($NH\mathbf{C}H_2$), 37.2 ($NH\mathbf{C}H_2$), 36.7 (DOTA with a weak signal), 35.4 ($CO\mathbf{C}H_2CH_2CH_2$), 33.3 ($COCHCH_2\mathbf{C}H_2CO$ of DUPA), 31.2 ($COCHCH_2\mathbf{C}H_2CO$ of DUPA), 31.1 ($COCHCH_2\mathbf{C}H_2CO$ of DUPA), 30.5 ($NHCH_2\mathbf{C}H_2CH_2O$), 30.3 ($NHCH_2\mathbf{C}H_2CH_2O$), 30.2 ($NHCH_2\mathbf{C}H_2CH_2O$), 30.1 ($COCH_2CH_2\mathbf{C}H_2$), 30.0 ($COCH\mathbf{C}H_2CH_2CO$ of DUPA), 29.9 (AMP), 28.8 ($COCH_2CH_2CH_2\mathbf{C}H_2$), 27.9 ($\mathbf{C}H_2CH_2NH$-DUPA), 27.1 ($COCH_2\mathbf{C}H_2CH_2$); MS (ESI-TOF) calcd for $C_{226}H_{389}N_{67}O_{66}$ 5097.9143, found 5100.5329 $(M + H)^+$.

G3-(DUPA)$_{16}$. A solution of 4-(aminomethyl)piperidine (0.19 g, 1.66 mmol) in THF (2 mL) was added to a solution of **5** (0.182 g, 8.65 μmol) in THF (3 mL). The solution was stirred at room temperature for 16 h and then evaporated under vacuum. The residue was dissolved in dichloromethane (30 mL), washed three times with brine, dried over $MgSO_4$, filtered, and

evaporated under vacuum. The resulting compound was dissolved in 3 mL of a mixture of TFA/TIPS/H_2O (95/2.5/2.5) and stirred at room temperature for 16 h. The solution was evaporated under vacuum. The residue was dissolved in methanol. The resulting solution was evaporated under vacuum to give **G3-(DUPA)$_{16}$** (0.17 g, quantitative)as a TFA salt. ^{1}H NMR (400 MHz, CD_3OD) δ 4.33-4.25 (br m, 32H, COC**H**CH_2CH_2CO of DUPA), 4.01-3.15 (m, 663H, C_3N_3-NHC**H$_2$**CH_2CH_2O, NC**H$_2$**C**H$_2$**N, C**H$_2$**OC**H$_2$**C**H$_2$**OC**H$_2$**C**H$_2$**OC**H$_2$**, CH_2C**H$_2$**NH, C**H**COO and C**H$_2$**COO of DOTA, AMP), 3.10-2.05 (m, 212H, COCHCH_2C**H$_2$**COO of DUPA, NHCOC**H$_2$**CH_2CH_2, COCHC**H$_2$**CH_2COO and COCHC**H$_2$**CH_2CONH of DUPA, DOTA, AMP), 1.91-1.73 (m, 172H, OCH_2C**H$_2$**CH_2NH, COCHCH_2C**H$_2$**CO of DUPA, AMP), 1.59 (br, 32H, COCH_2C**H$_2$**CH_2), 1.49 (br, 32H, CH_2CH_2C**H$_2$**CH_2NH), 1.39-1.23 (br m, 144H, C**H$_2$**C**H$_2$**C**H$_2$**CH_2CH_2NH-DUPA, AMP); ^{13}C NMR (100 MHz, CD_3OD) δ 176.5 (**C**OO), 176.4 (**C**OO), 176.0 (**C**OO), 175.9 (**C**OO), 175.0 (NH**C**OCH_2), 174.8 (NH**C**OCH_2), 163.3 (**C**$_3N_3$), 163.0 (**C**$_3N_3$), 160.2 (NH**C**ONH of DUPA), 157.2 (NH**C**ONH of DUPA), 71.6 (two lines, O**C**H$_2$**C**H$_2$O), 71.3 (two lines, O**C**H$_2$CH$_2$O), 70.0 (O**C**H$_2$**C**H$_2$O), 69.8 (br, O**C**H$_2$**C**H$_2$O, NHCH_2CH_2**C**H$_2$O), 68.9 (NHCH_2CH_2**C**H$_2$O), 55.9 (DOTA with a weak signal), 53.9 (CO**C**HCH_2CH_2CO of DUPA), 53.8 (CO**C**HCH_2CH_2CO of DUPA), 52.9 (DOTA with a weak signal), 52.4 (DOTA with a weak signal), 45.4 (br, AMP), 44.9 (br, piperazine), 43.9 (AMP), 40.6 (NH**C**H$_2$), 39.7 (br, NH**C**H$_2$), 39.3 (AMP), 37.9 (NH**C**H$_2$), 37.2 (NH**C**H$_2$), 36.7 (DOTA with a weak signal), 35.4 (CO**C**H$_2CH_2CH_2$), 33.3 (COCHCH_2**C**H$_2$CO of DUPA), 31.2 (COCHCH_2**C**H$_2$CO of DUPA), 31.1 (COCHCH_2**C**H$_2$CO of DUPA), 30.5 (NHCH_2**C**H$_2CH_2$O), 30.3 (NHCH_2**C**H$_2CH_2$O), 30.2 (NHCH_2**C**H$_2CH_2$O), 30.1 (COCH_2CH_2**C**H$_2$), 30.0 (COCH**C**H$_2CH_2$CO of DUPA), 29.9 (AMP), 28.8 (CO$CH_2CH_2CH_2$**C**H$_2$), 27.9 (**C**H$_2CH_2$NH-DUPA), 27.0 (COCH_2**C**H$_2CH_2$); MS (ESI-TOF) calcd for $C_{862}H_{1473}N_{271}O_{234}$ 19366.17, found 19377.02 $(M + H)^+$.

G5-(DUPA)$_{64}$. A solution of 4-(aminomethyl)piperidine (0.13 g, 1.14 mmol) in THF (2 mL) was added to a solution of **6** (0.122 g, 1.48 μmol) in THF (3 mL). The solution was stirred at room temperature for 16 h and then evaporated under vacuum. The residue was dissolved in dichloromethane (30 mL), washed three times with brine, dried over $MgSO_4$, filtered, and evaporated under vacuum. The resulting compound was dissolved in 3 mL of a mixture of TFA/TIPS/H_2O (95/2.5/2.5) and stirred at room temperature for 16 h. The solution was evaporated under vacuum. The residue was dissolved in methanol. The resulting solution was evaporated under vacuum to give **G5-(DUPA)$_{64}$** (0.12 g, quantitative) as a TFA salt. ^{1}H NMR (400 MHz, CD_3OD) δ 4.34-4.25 (br m, 128H, $COC\mathbf{H}CH_2CH_2CO$ of DUPA), 4.01-3.15 (m, 2640H, C_3N_3-$NHC\mathbf{H_2}CH_2CH_2O$, $NC\mathbf{H_2}C\mathbf{H_2}N$, $C\mathbf{H_2}OC\mathbf{H_2}C\mathbf{H_2}OC\mathbf{H_2}C\mathbf{H_2}OC\mathbf{H_2}$, $CH_2C\mathbf{H_2}NH$, $C\mathbf{H}COO$ and $C\mathbf{H_2}COO$ of DOTA, AMP), 3.11-2.06 (m, 788H, $COCHCH_2C\mathbf{H_2}COO$ of DUPA, $NHCOC\mathbf{H_2}CH_2CH_2$, $COCHC\mathbf{H_2}CH_2COO$ and $COCHC\mathbf{H_2}CH_2CONH$ of DUPA, DOTA, AMP), 1.95-1.72 (m, 684H, $OCH_2C\mathbf{H_2}CH_2NH$, $COCHCH_2C\mathbf{H_2}CO$ of DUPA, AMP), 1.59 (br, 128H, $COCH_2C\mathbf{H_2}CH_2$), 1.49 (br, 128H, $CH_2CH_2C\mathbf{H_2}CH_2NH$), 1.49-1.24 (br m, 576H, $C\mathbf{H_2}C\mathbf{H_2}C\mathbf{H_2}CH_2CH_2NH$-DUPA, AMP); ^{13}C NMR (100 MHz, CD_3OD) δ 176.5 (two lines, $NH\mathbf{C}OCH_2$), 176.4 ($\mathbf{C}OO$), 176.0 ($\mathbf{C}OO$), 175.9 ($\mathbf{C}OO$), 175.0 ($NH\mathbf{C}OCH_2$), 174.8 ($NH\mathbf{C}OCH_2$), 174.7 ($NH\mathbf{C}OCH_2$), 164.0 ($\mathbf{C}_3N_3$), 160.2 ($NH\mathbf{C}ONH$ of DUPA), 157.3 ($NH\mathbf{C}ONH$ of DUPA), 71.6 ($O\mathbf{C}H_2\mathbf{C}H_2O$), 71.3 (two lines, $O\mathbf{C}H_2\mathbf{C}H_2O$), 70.0 ($O\mathbf{C}H_2\mathbf{C}H_2O$), 69.9 (br, $O\mathbf{C}H_2\mathbf{C}H_2O$), 69.8 (br, $NHCH_2CH_2\mathbf{C}H_2O$), 69.0 ($NHCH_2CH_2\mathbf{C}H_2O$), 55.9 (DOTA), 53.9 ($CO\mathbf{C}HCH_2CH_2CO$ of DUPA), 53.8 ($CO\mathbf{C}HCH_2CH_2CO$ of DUPA), 45.5 (br, AMP), 45.0 (br, piperazine), 43.9 (AMP), 40.6 ($NH\mathbf{C}H_2$), 39.7 (br, $NH\mathbf{C}H_2$, AMP), 37.9 ($NH\mathbf{C}H_2$), 37.2 ($NH\mathbf{C}H_2$), 36.7 (DOTA with a weak signal), 35.4 ($CO\mathbf{C}H_2CH_2CH_2$), 33.3 ($COCHCH_2\mathbf{C}H_2CO$ of DUPA), 31.2 ($COCHCH_2\mathbf{C}H_2CO$ of DUPA), 31.1 ($COCHCH_2\mathbf{C}H_2CO$ of DUPA), 30.5 ($NHCH_2\mathbf{C}H_2CH_2O$),

30.4 ($NHCH_2\mathbf{C}H_2CH_2O$), 30.2 ($NHCH_2\mathbf{C}H_2CH_2O$), 30.1 (br, $COCH_2CH_2\mathbf{C}H_2$, $COCH\mathbf{C}H_2CH_2CO$ of DUPA), 29.6 (AMP), 28.9 ($COCH_2CH_2CH_2\mathbf{C}H_2$), 27.9 ($\mathbf{C}H_2CH_2NH$-DUPA), 27.1 ($COCH_2\mathbf{C}H_2CH_2$).

Spectra

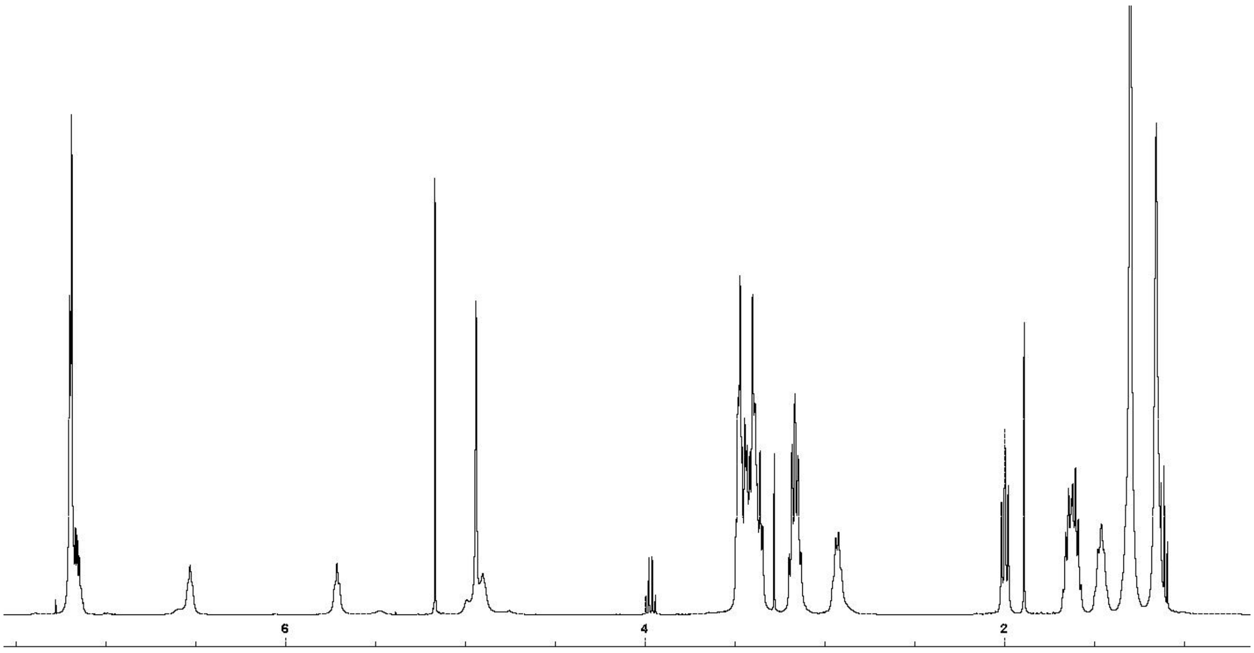

Figure S1. ^{1}H NMR spectrum of **1** (400 MHz, $CDCl_3$).

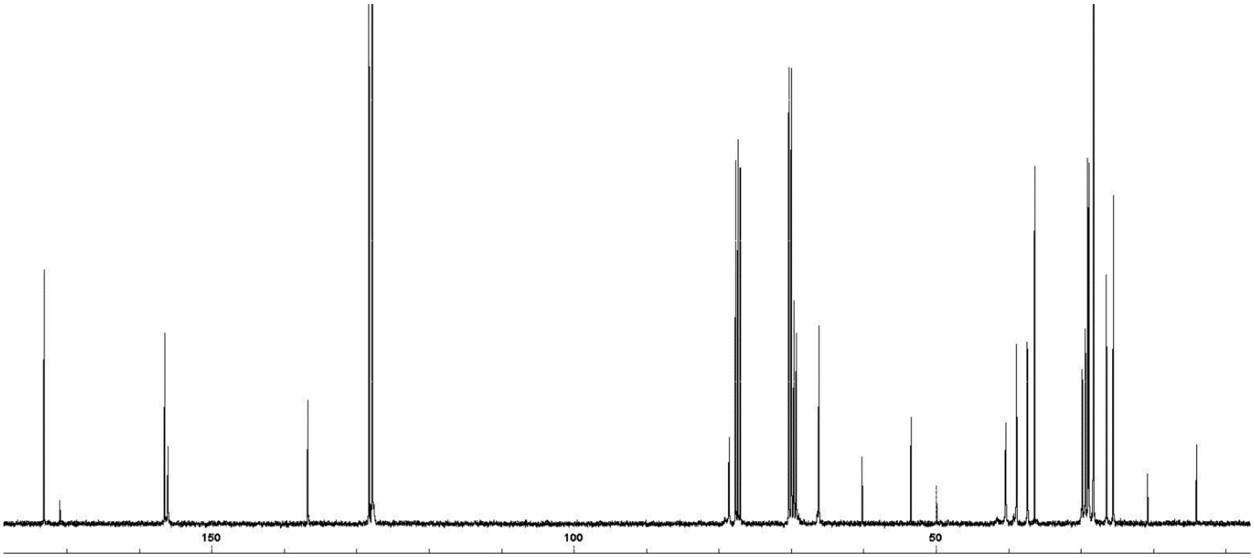

Figure S2. ^{13}C NMR spectrum of **1** (100 MHz, $CDCl_3$).

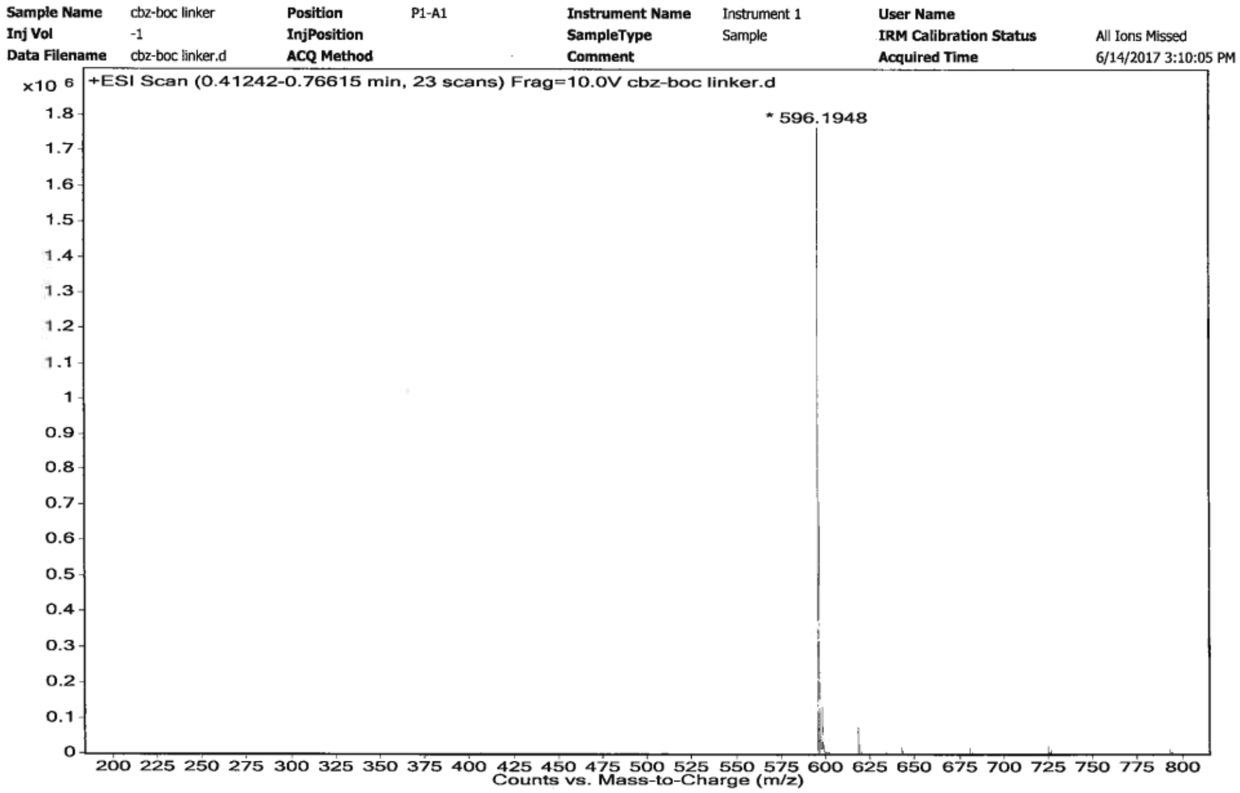

Figure S3. ESI-TOF mass spectrum of **1**.

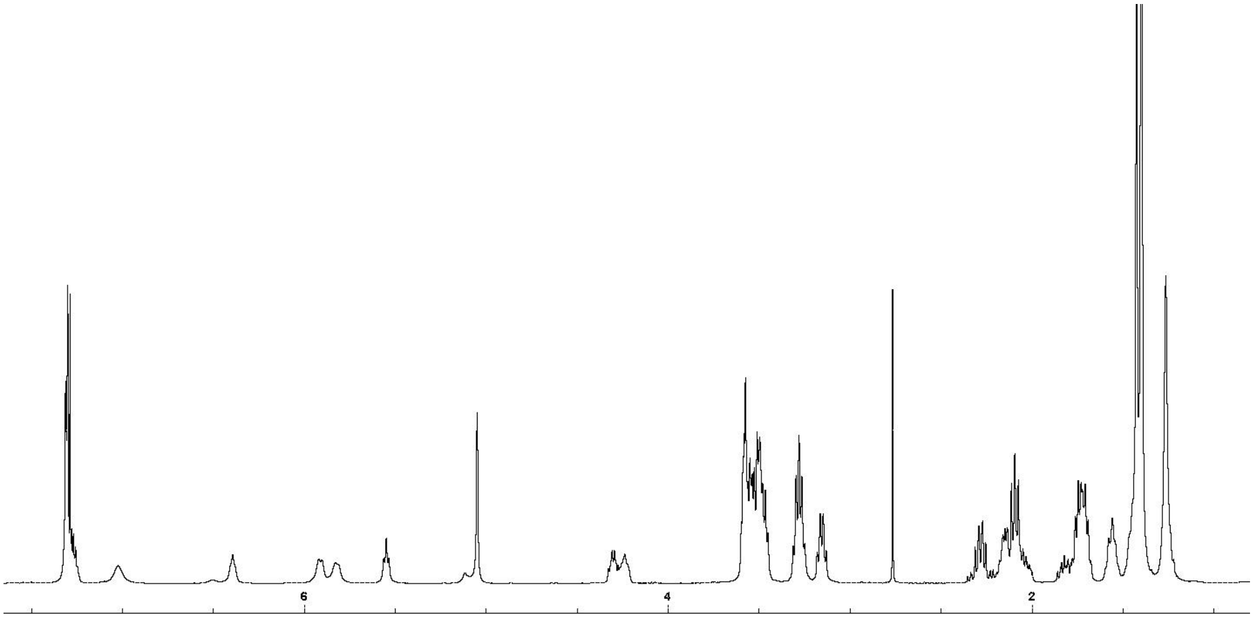

Figure S4. ^{1}H NMR spectrum of **2** (400 MHz, $CDCl_3$).

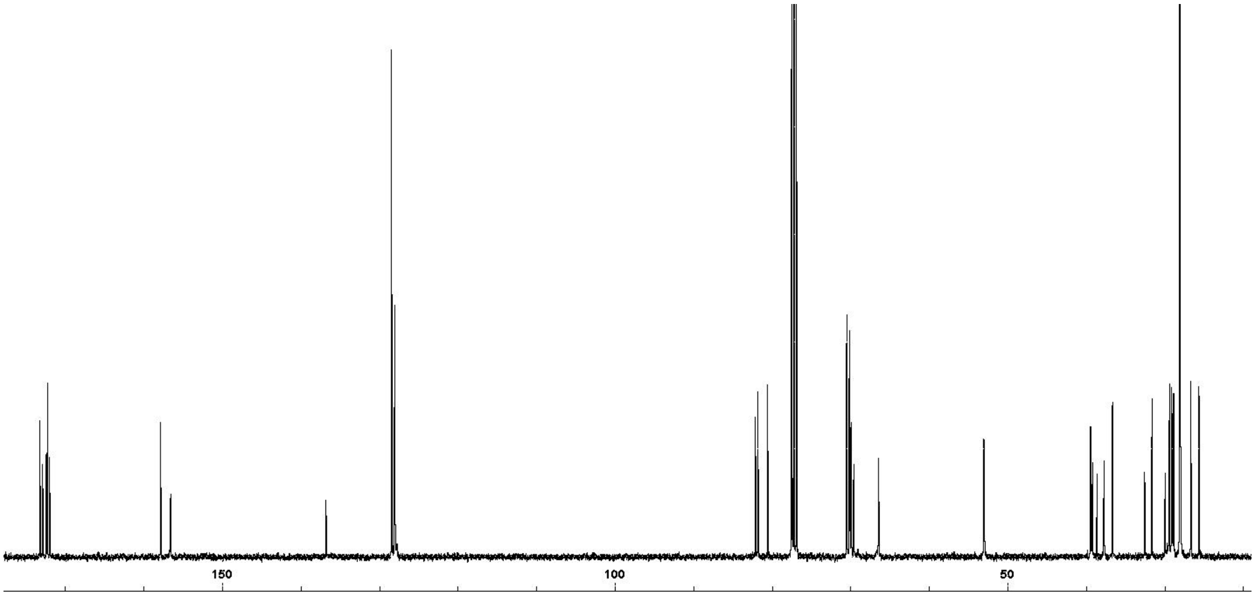

Figure S5. ^{13}C NMR spectrum of **2** (100 MHz, $CDCl_3$).

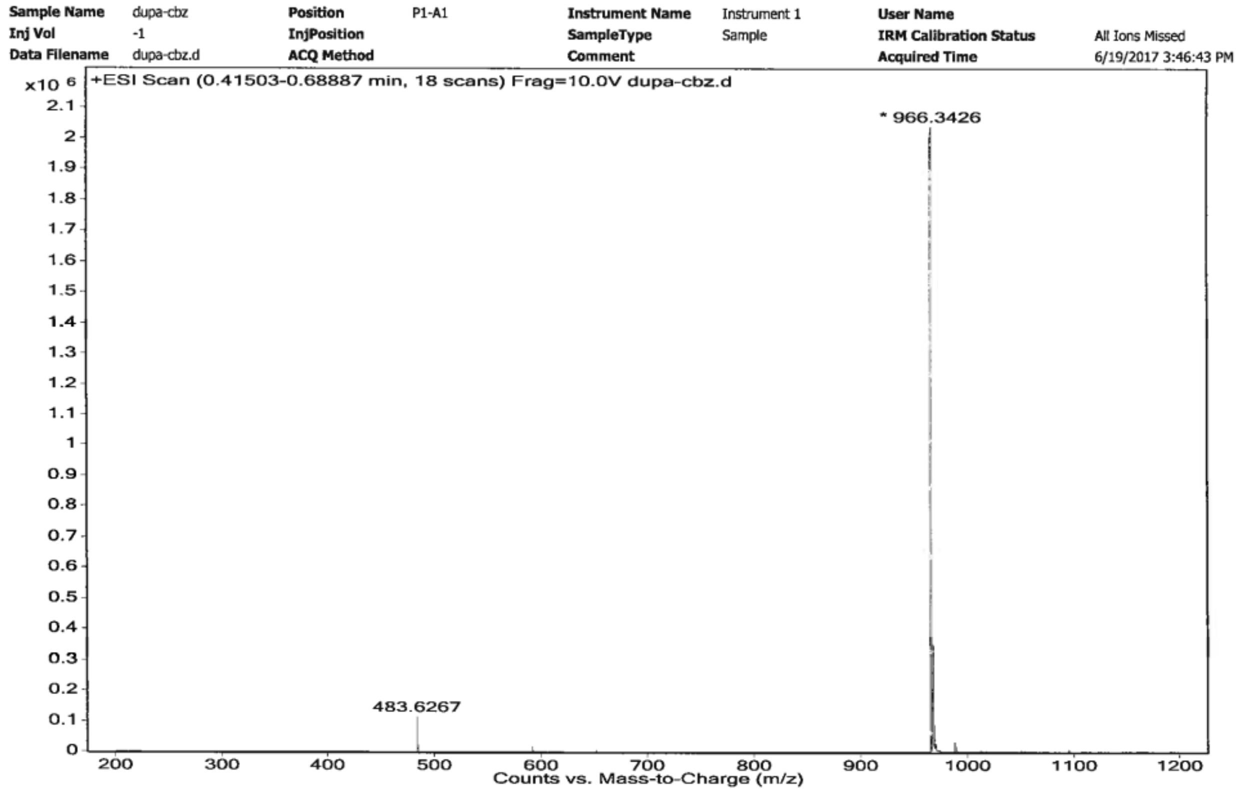

Figure S6. ESI-TOF mass spectrum of **2**.

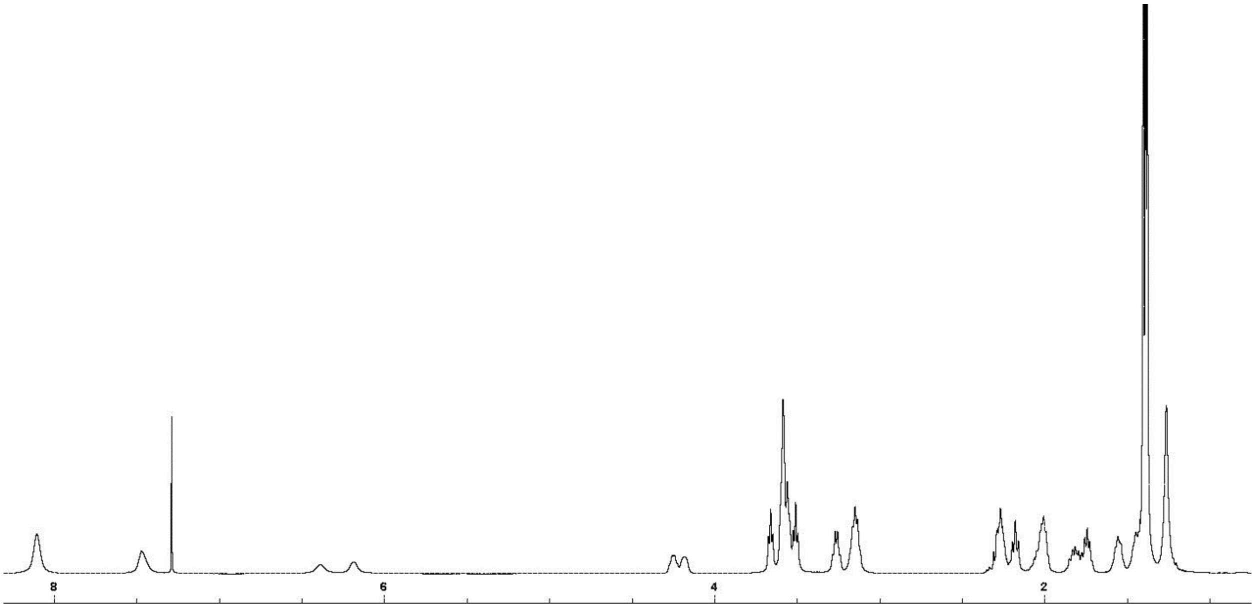

Figure S7. ^{1}H NMR spectrum of **3** (400 MHz, $CDCl_3$).

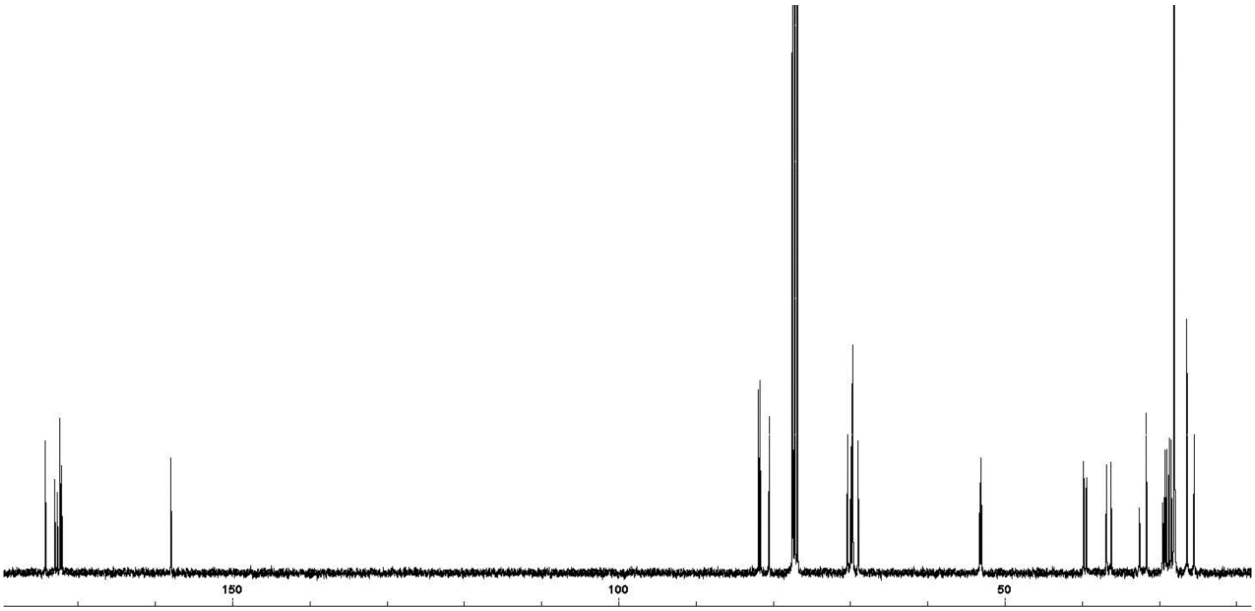

Figure S8. ^{13}C NMR spectrum of **3** (100 MHz, $CDCl_3$).

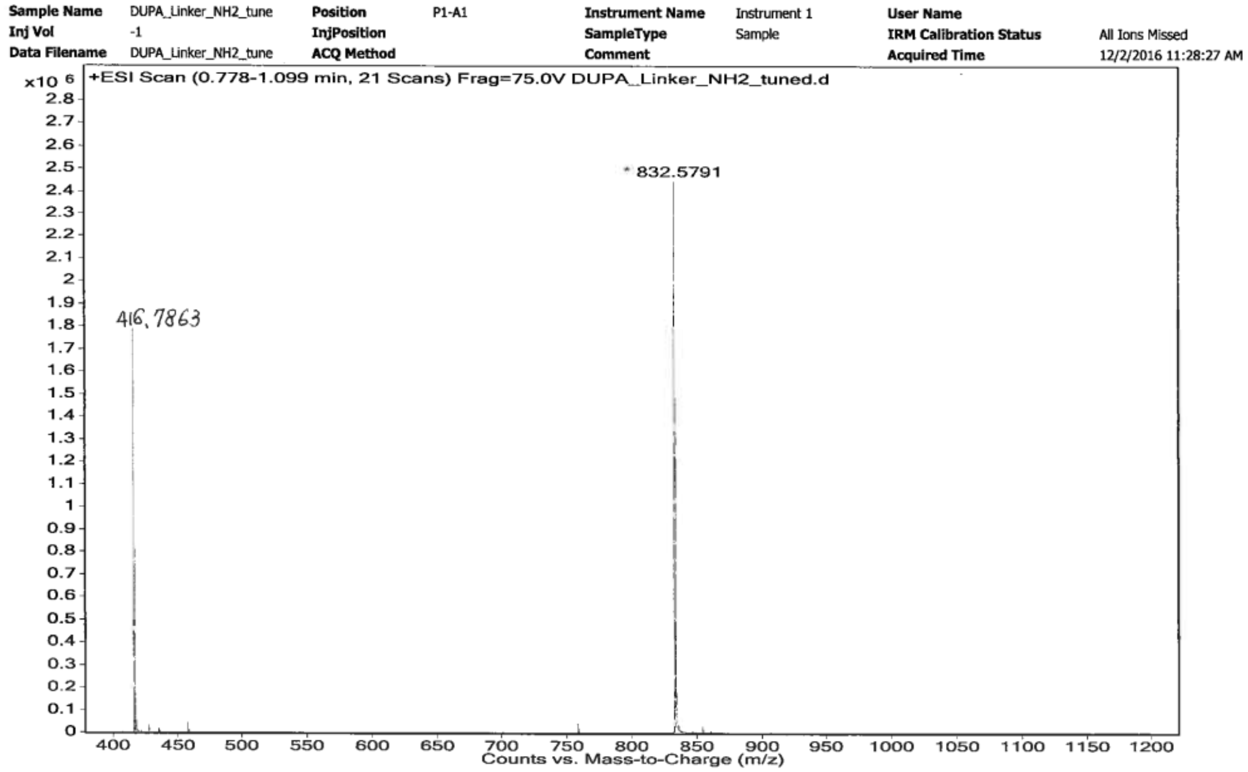

Figure S9. ESI-TOF mass spectrum of **3**.

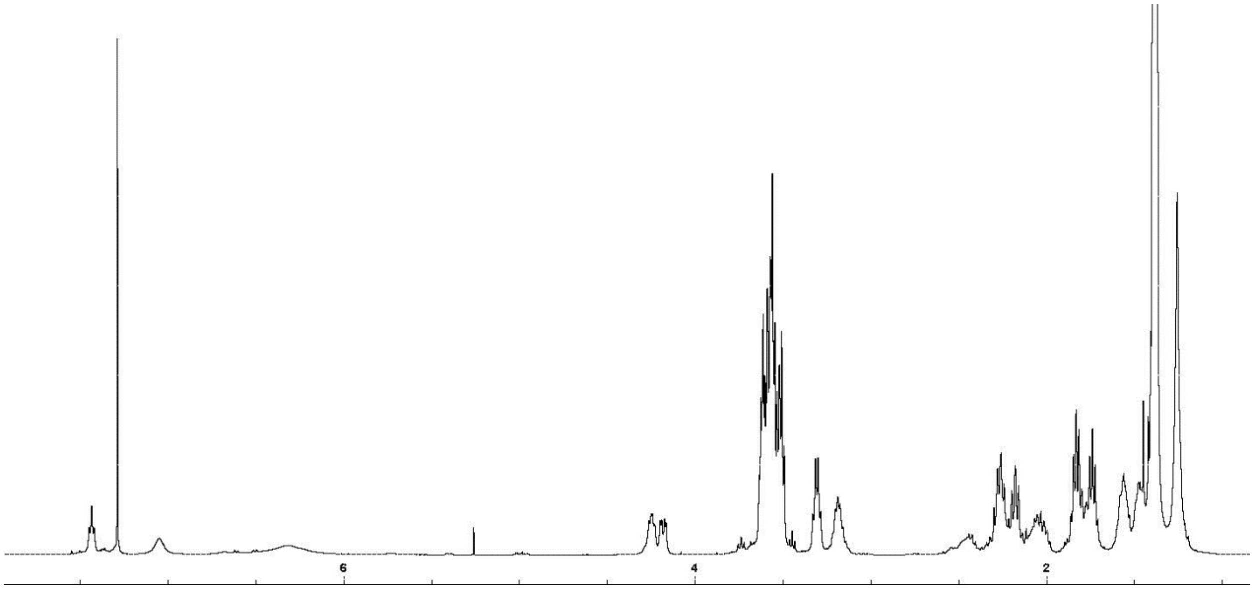

Figure S10. ^{1}H NMR spectrum of **DUPA-DCT** (400 MHz, $CDCl_3$).

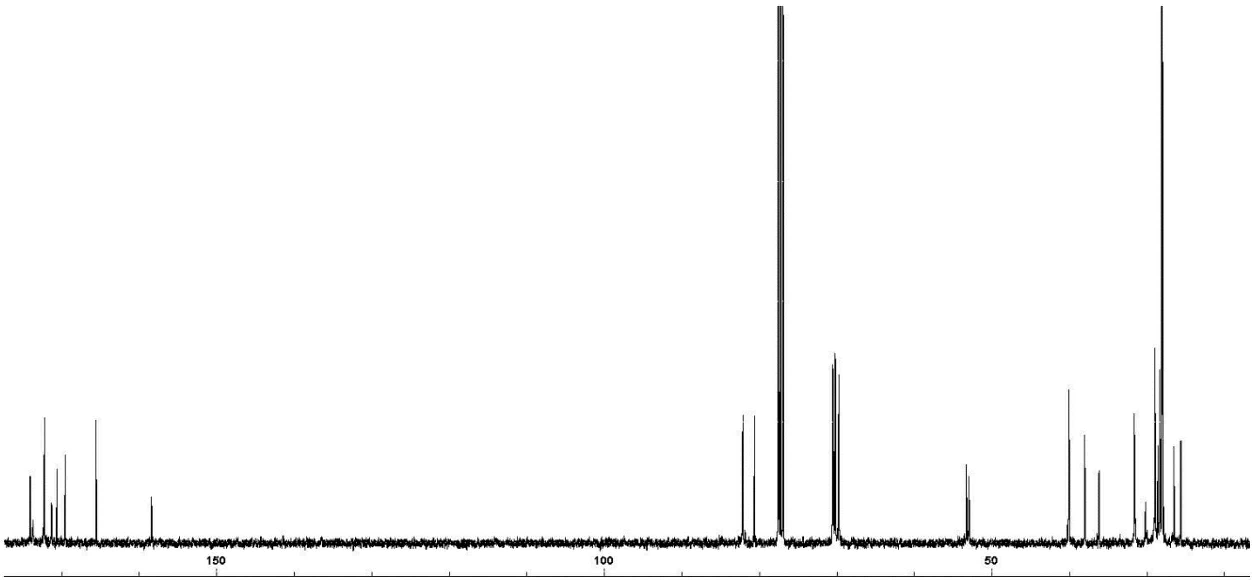

Figure S11. ^{13}C NMR spectrum of **DUPA-DCT** (100 MHz, $CDCl_3$).

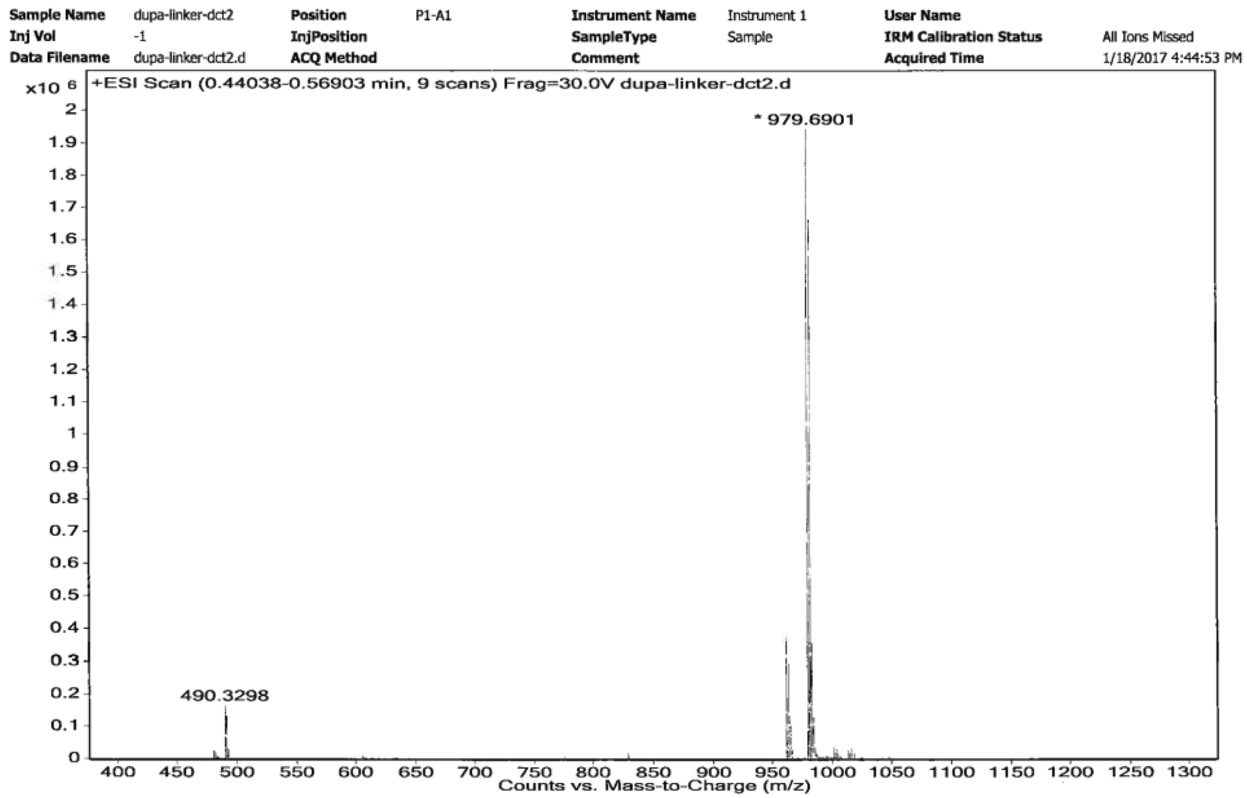

Figure S12. ESI-TOF mass spectrum of **DUPA-DCT**.

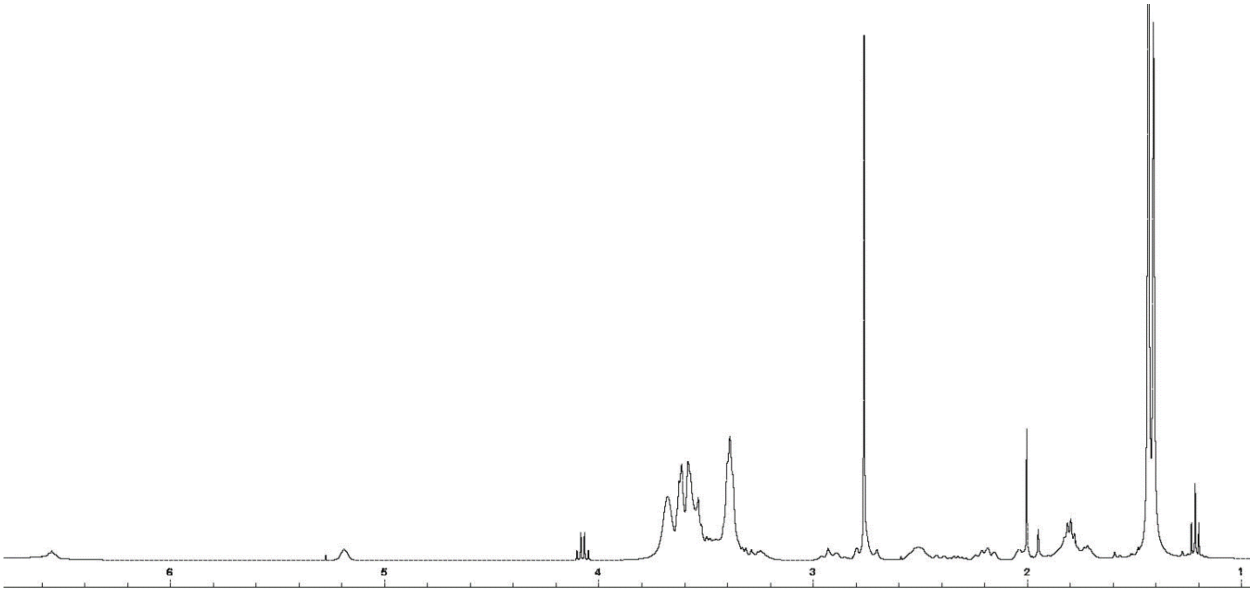

Figure S13. ^{1}H NMR spectrum of **G1 Platform** (400 MHz, $CDCl_3$).

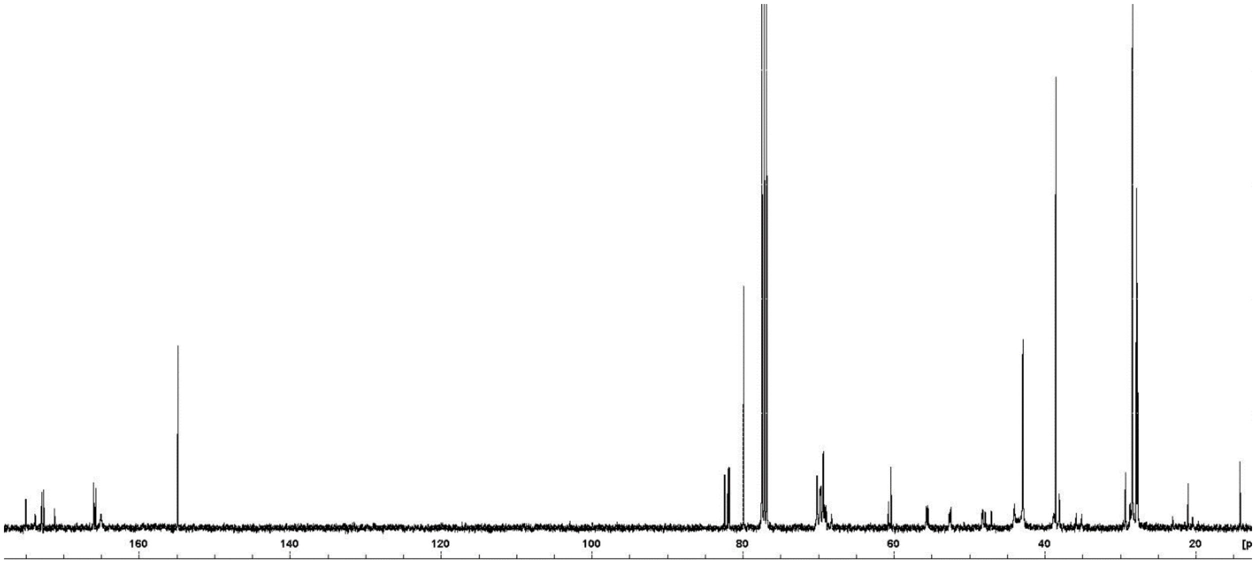

Figure S14. ^{13}C NMR spectrum of **G1 Platform** (100 MHz, $CDCl_3$).

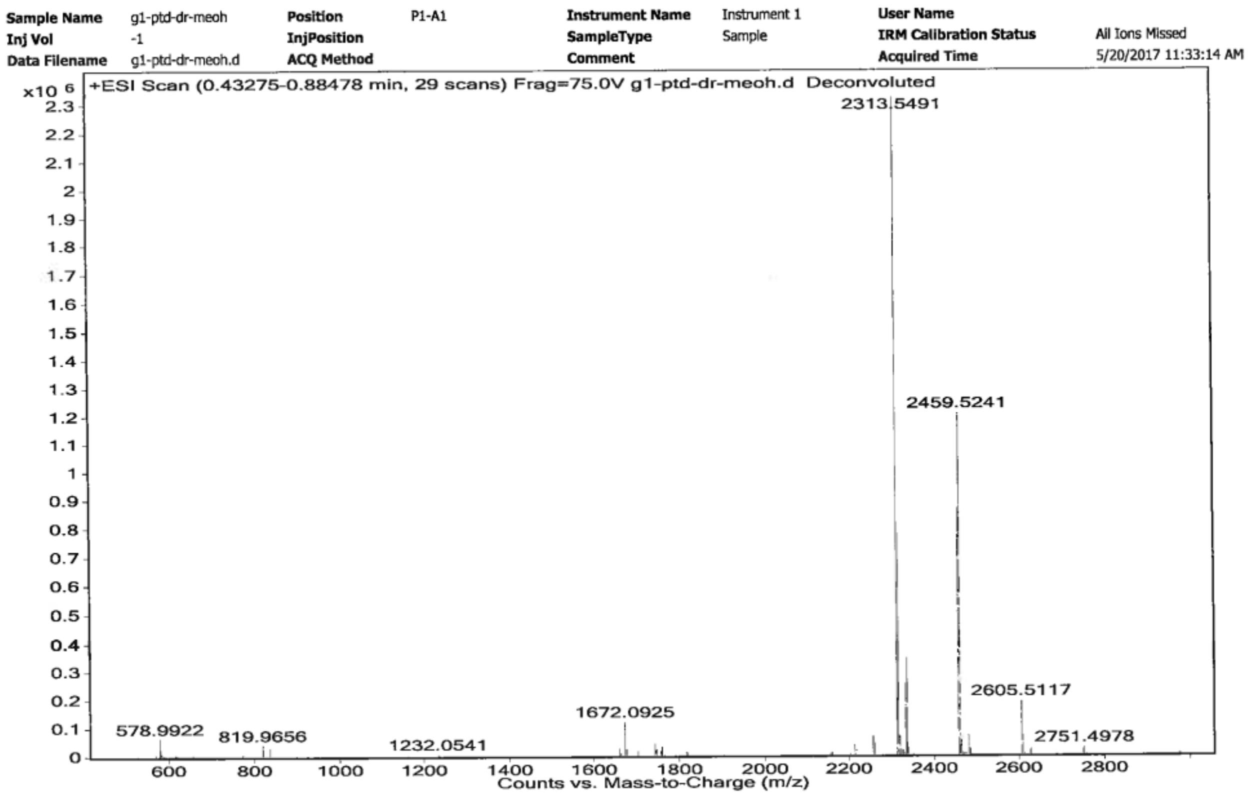

Figure S15. ESI-TOF mass spectrum of **G1 Platform**.

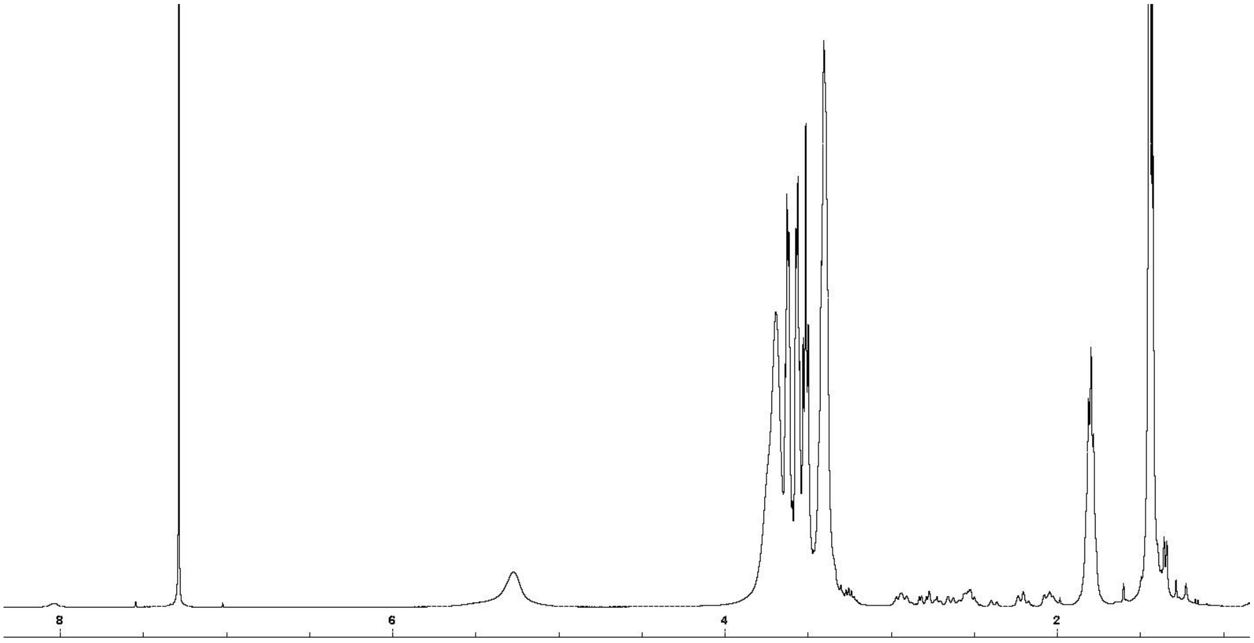

Figure S16. ^{1}H NMR spectrum of **G3 Platform** (400 MHz, $CDCl_3$).

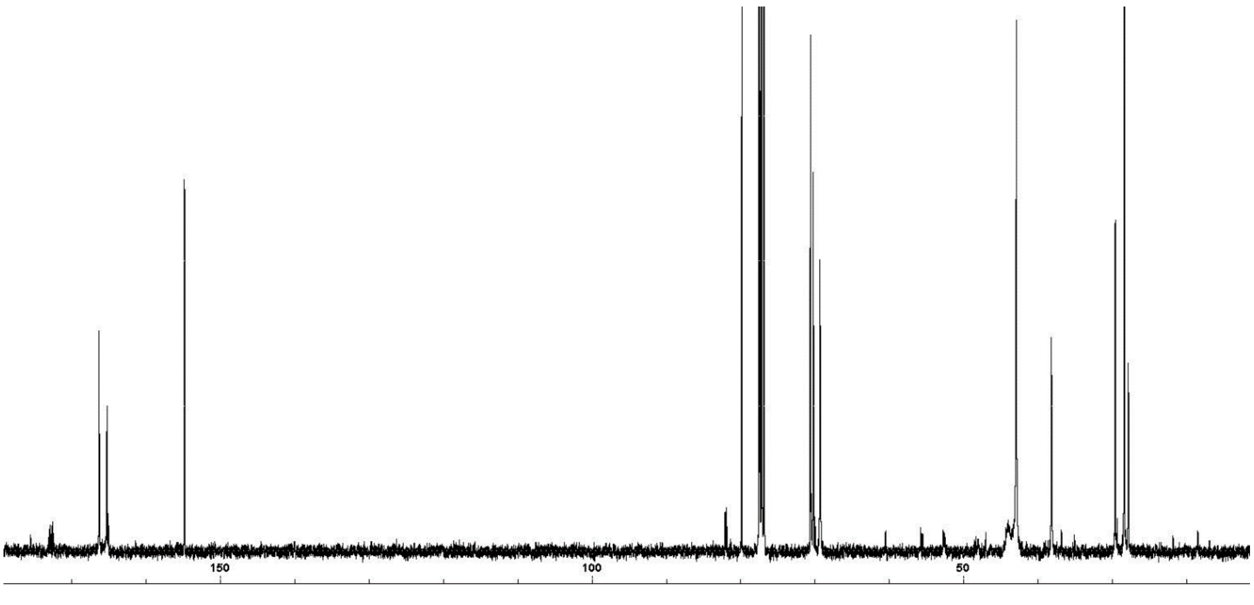

Figure S17. ^{13}C NMR spectrum of **G3 Platform** (100 MHz, $CDCl_3$).

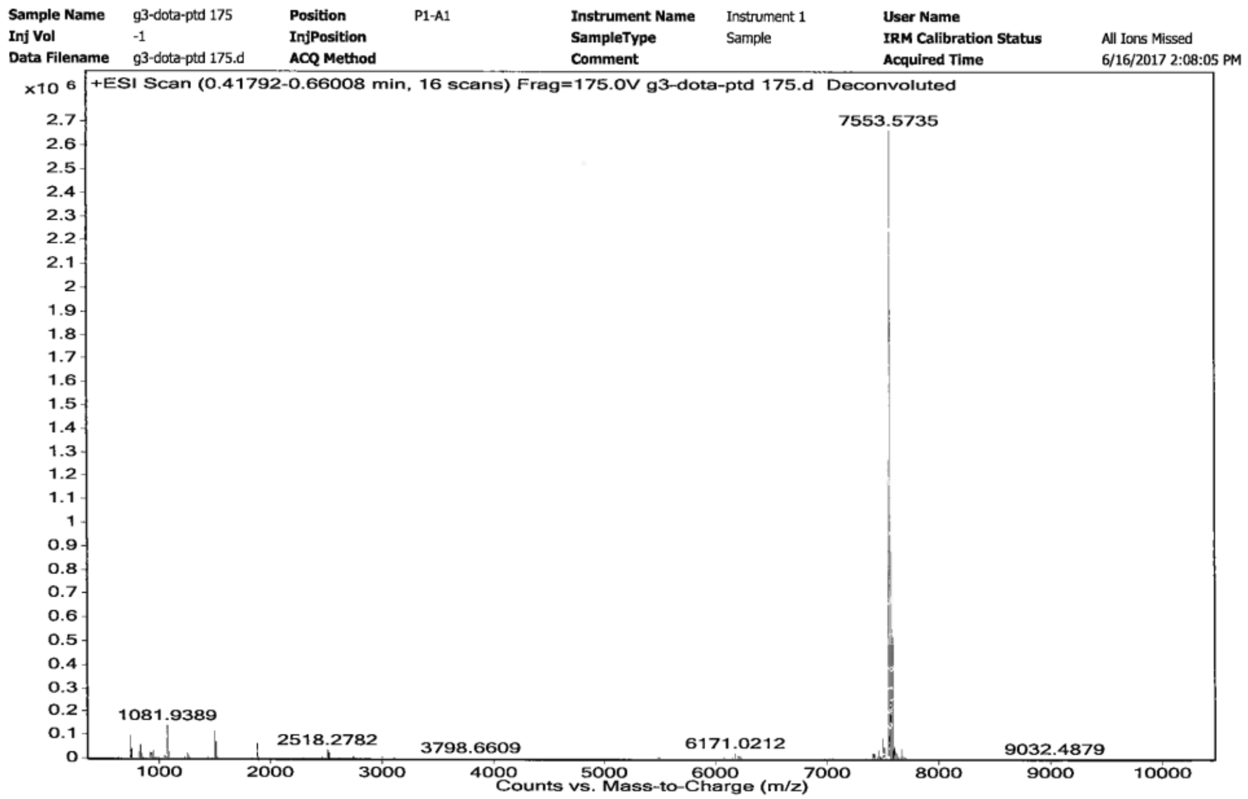

Figure S18. ESI-TOF mass spectrum of **G3 Platform**.

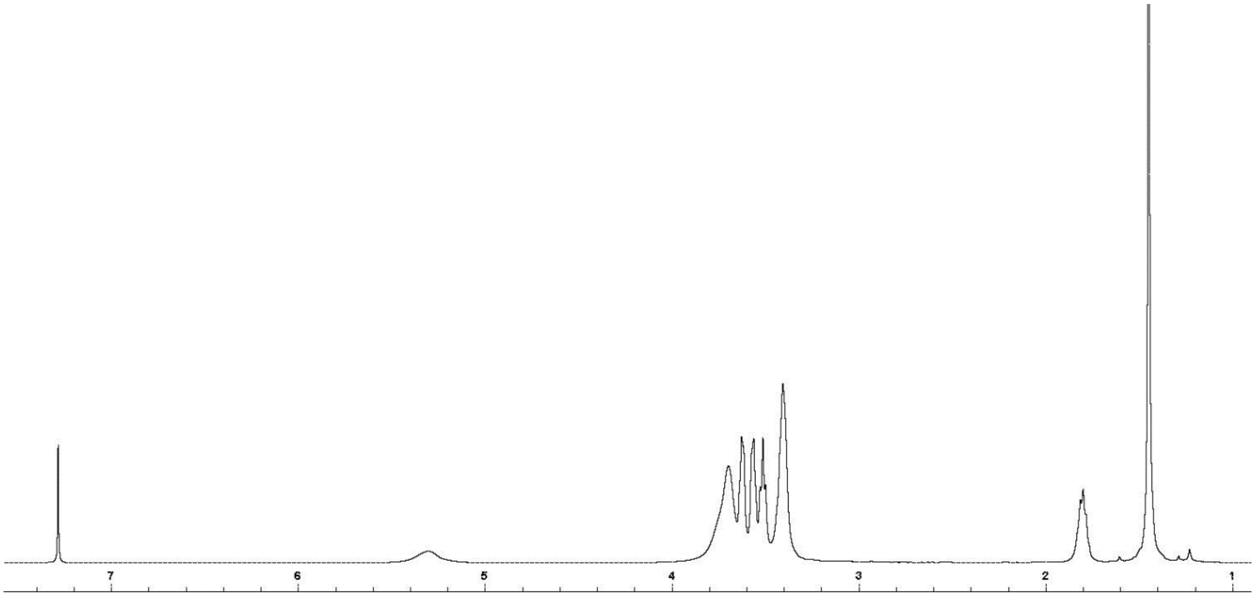

Figure S19. ^{1}H NMR spectrum of **G5 Platform** (400 MHz, $CDCl_3$).

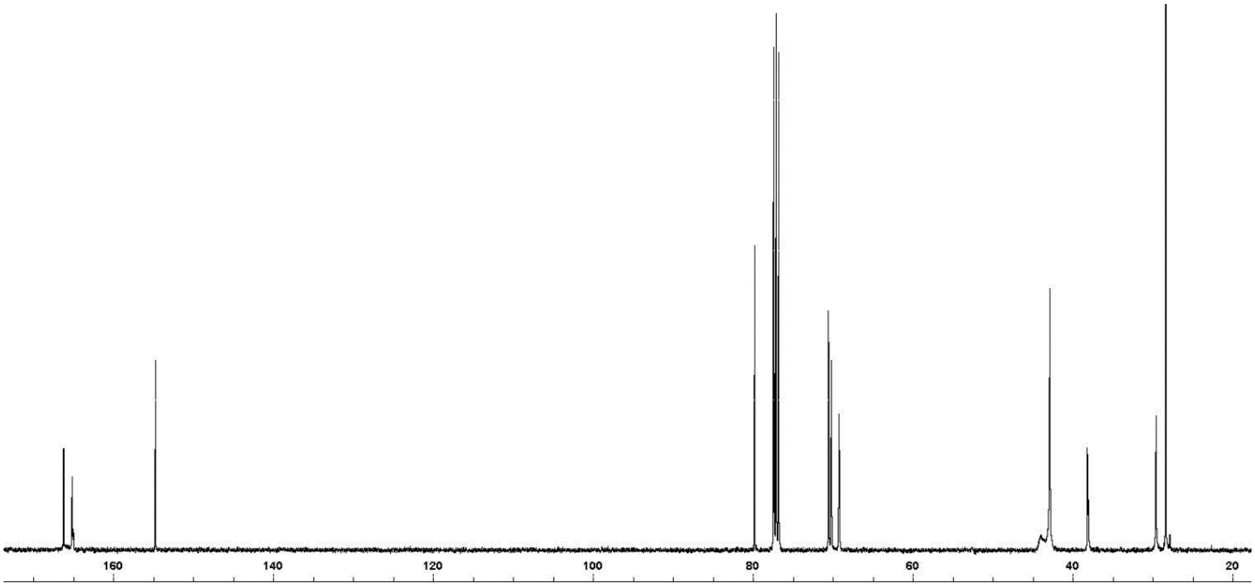

Figure S20. ^{13}C NMR spectrum of **G5 Platform** (100 MHz, $CDCl_3$).

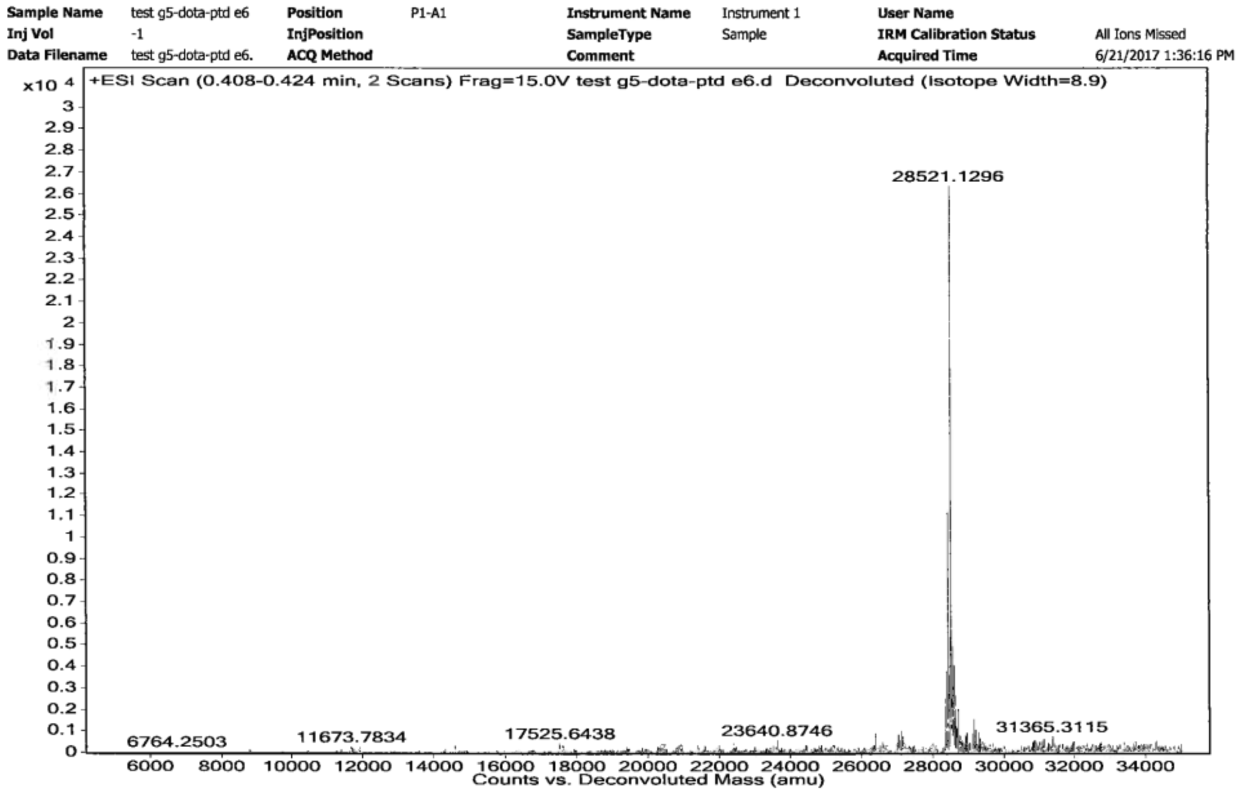

Figure S21. ESI-TOF mass spectrum of **G5 Platform**.

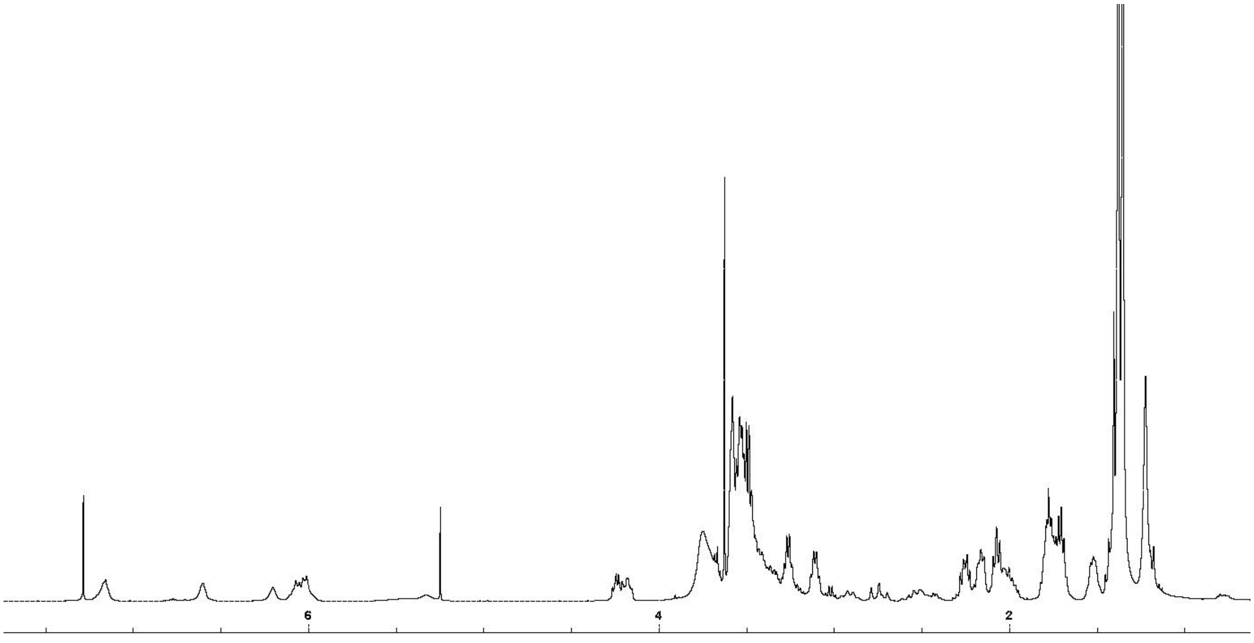

Figure S22. ^{1}H NMR spectrum of **4** (400 MHz, $CDCl_3$).

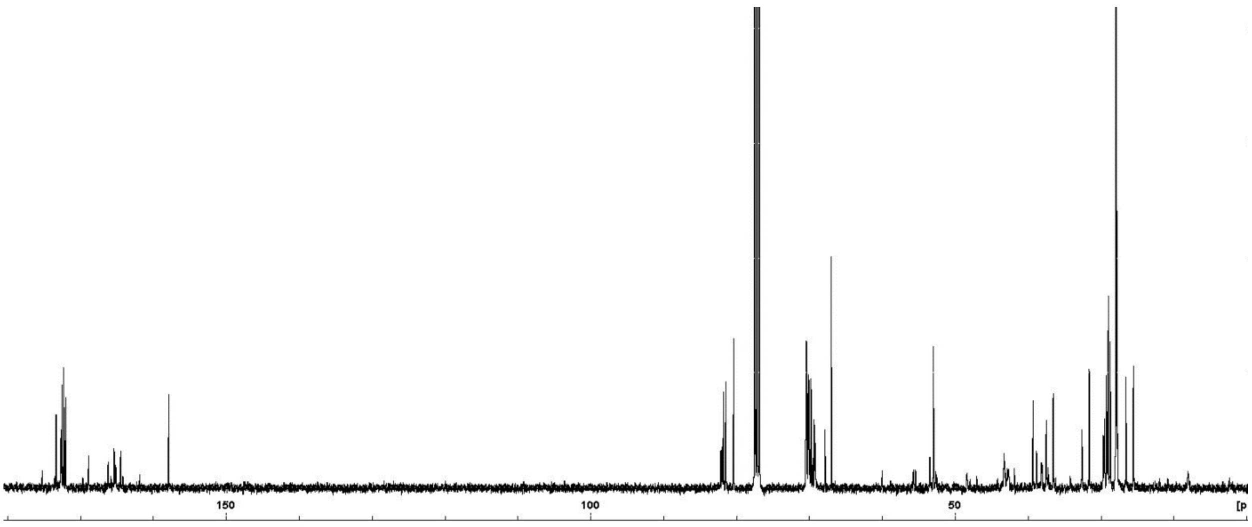

Figure S23. ^{13}C NMR spectrum of **4** (100 MHz, $CDCl_3$).

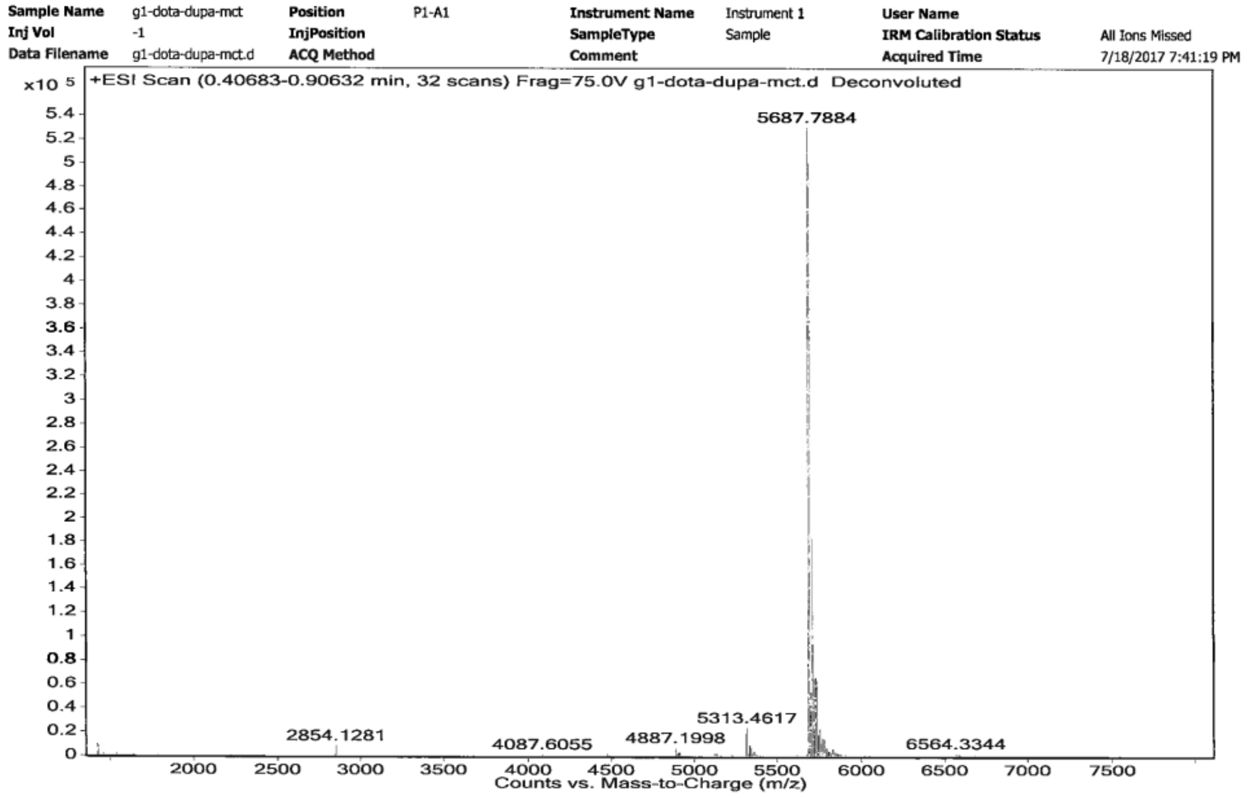

Figure S24. ESI-TOF mass spectrum of **4**.

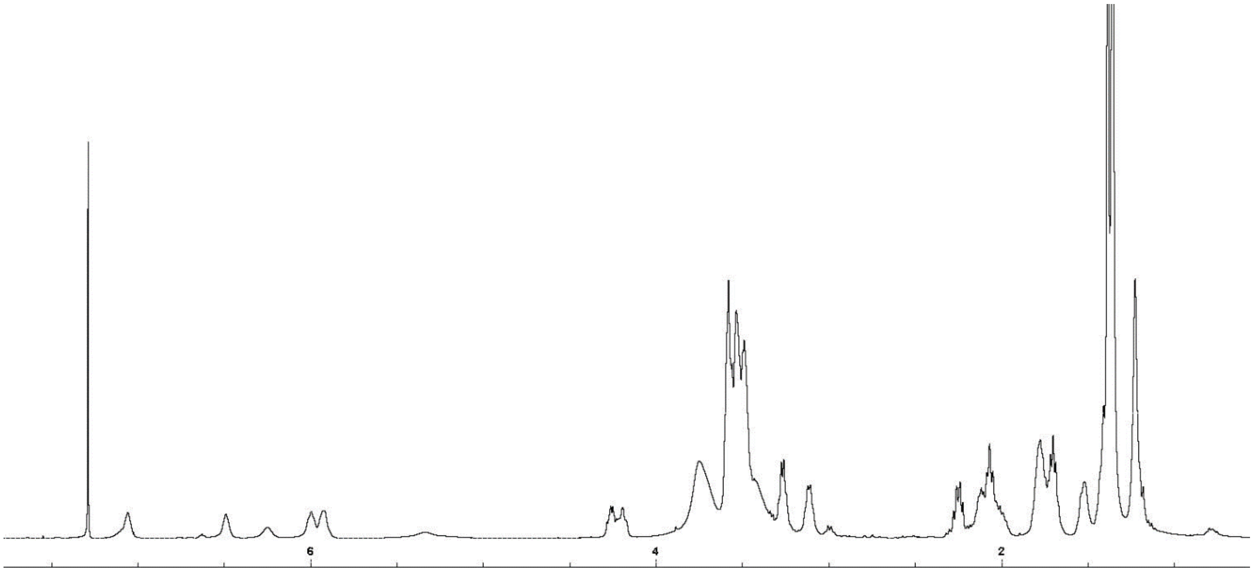

Figure S25. ^{1}H NMR spectrum of **5** (400 MHz, $CDCl_3$).

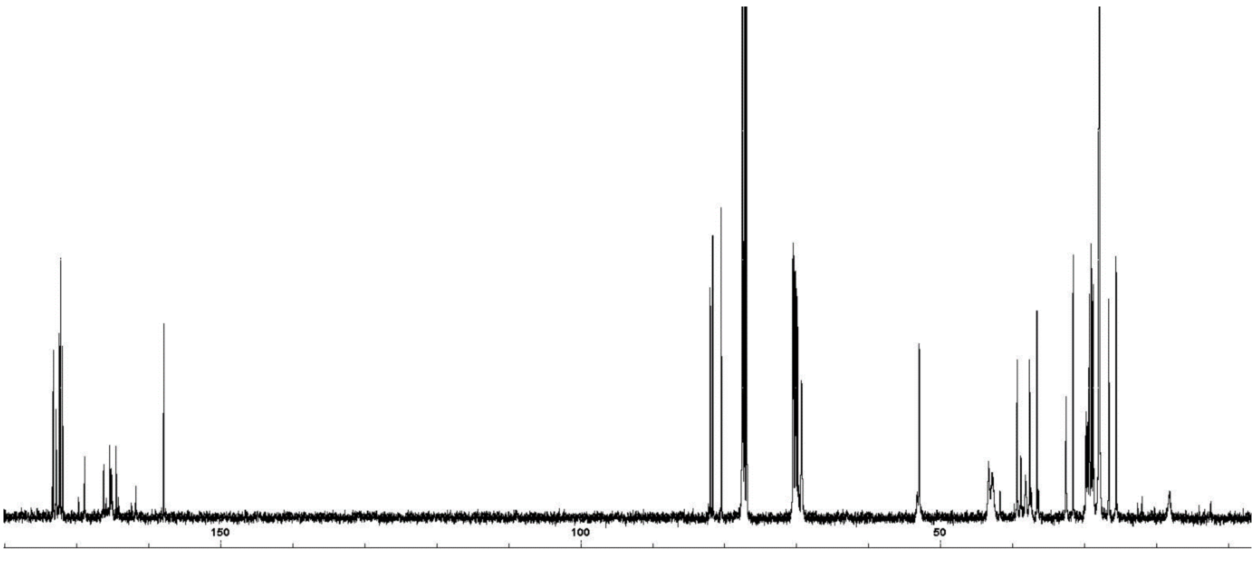

Figure S26. ^{13}C NMR spectrum of **5** (100 MHz, $CDCl_3$).

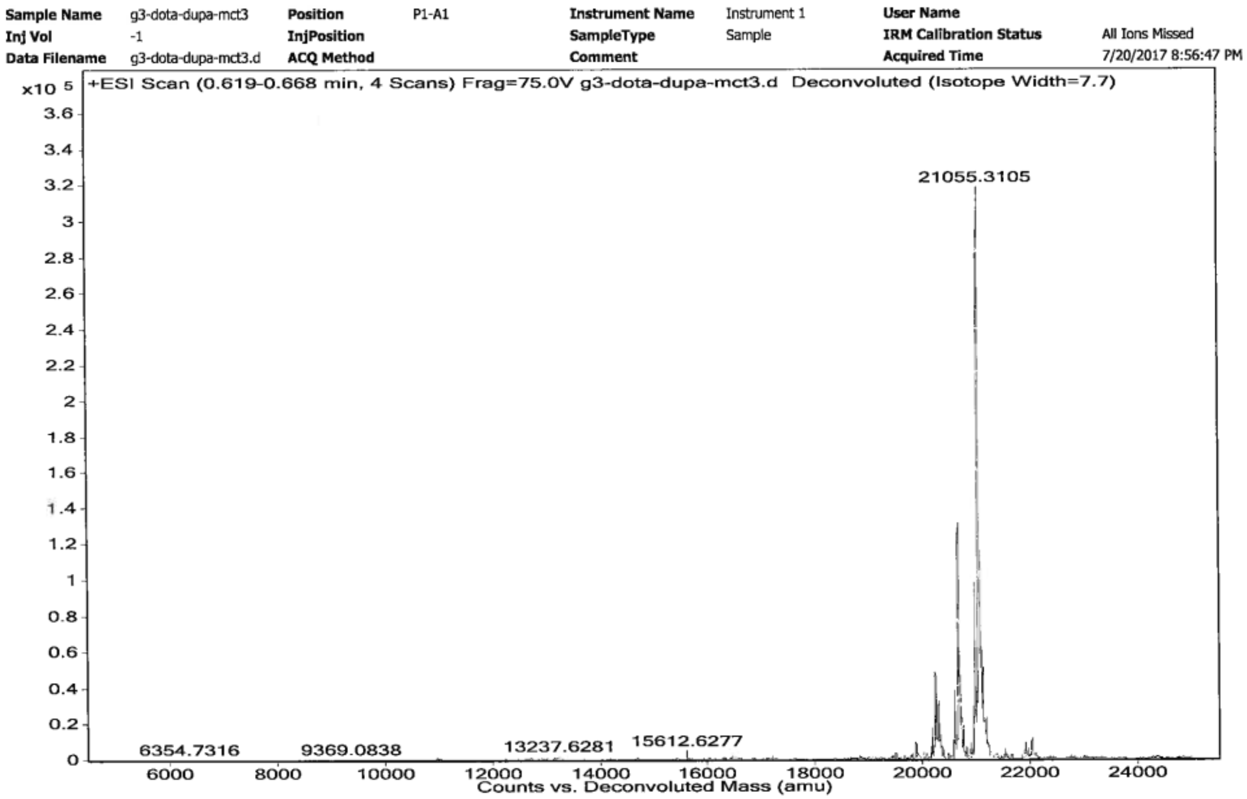

Figure S27 . ESI-TOF mass spectrum of **5**.

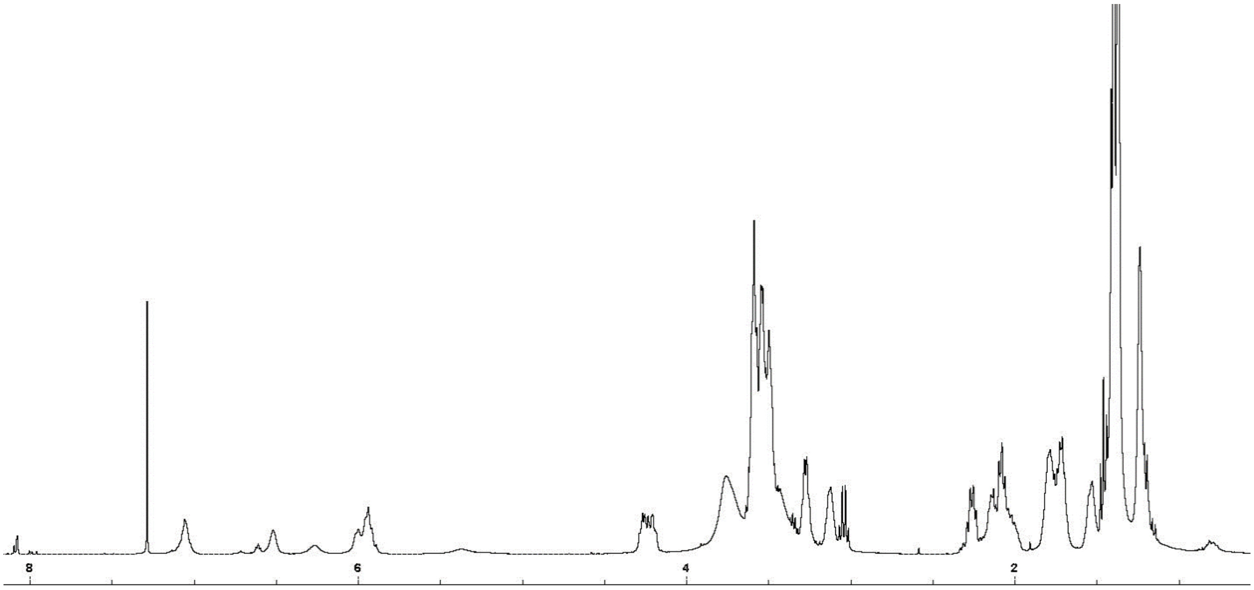

Figure S28. ^{1}H NMR spectrum of **6** (400 MHz, $CDCl_3$).

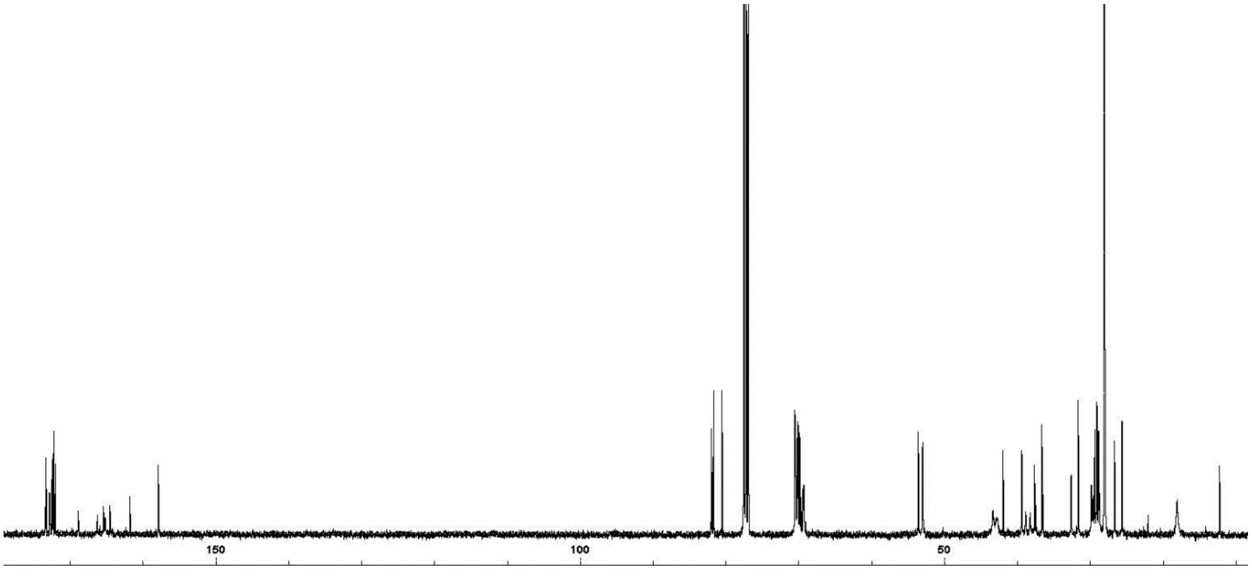

Figure S29. ^{13}C NMR spectrum of **6** (100 MHz, $CDCl_3$).

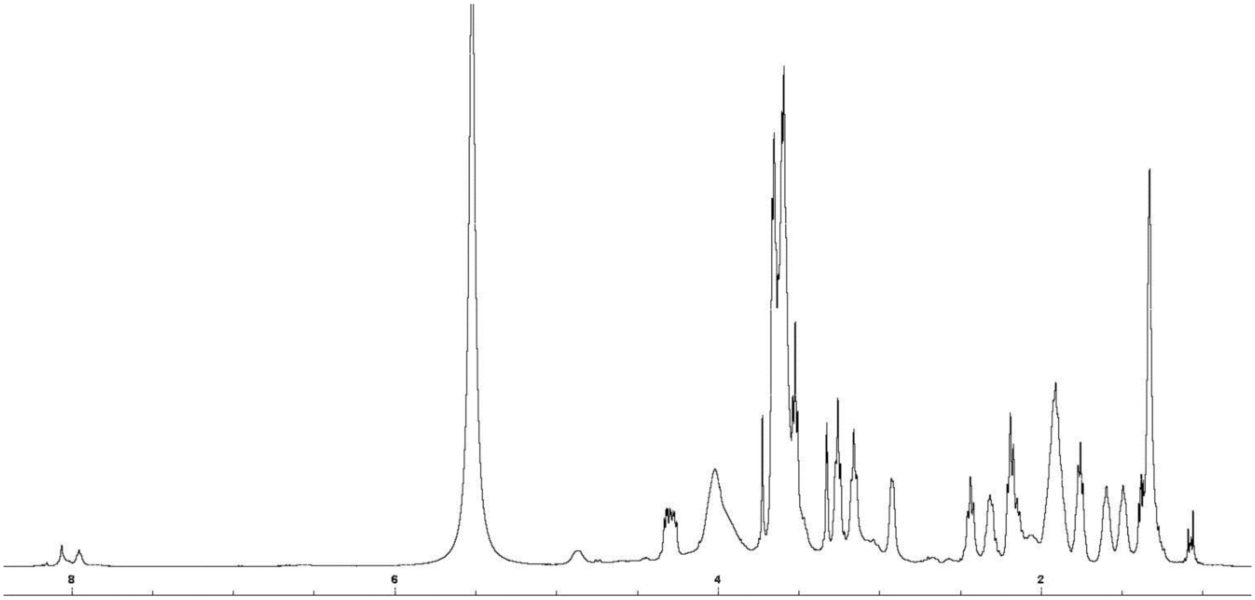

Figure S30. ^{1}H NMR spectrum of **G1-(DUPA)$_4$** (400 MHz, CD_3OD).

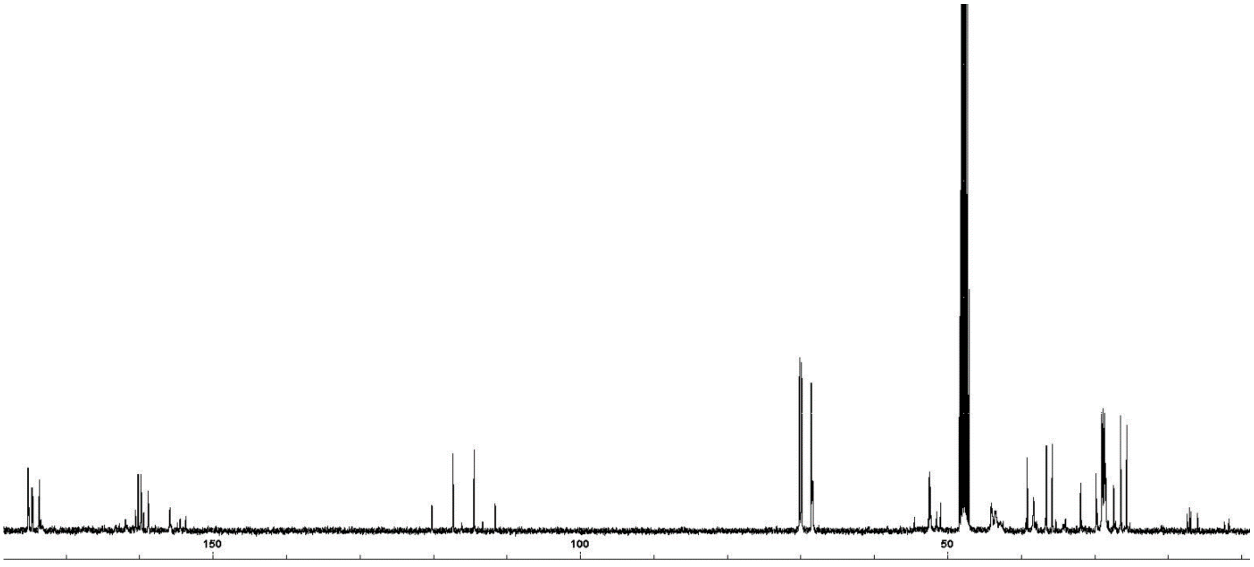

Figure S31. ^{13}C NMR spectrum of **G1-(DUPA)$_4$** (100 MHz, CD_3OD).

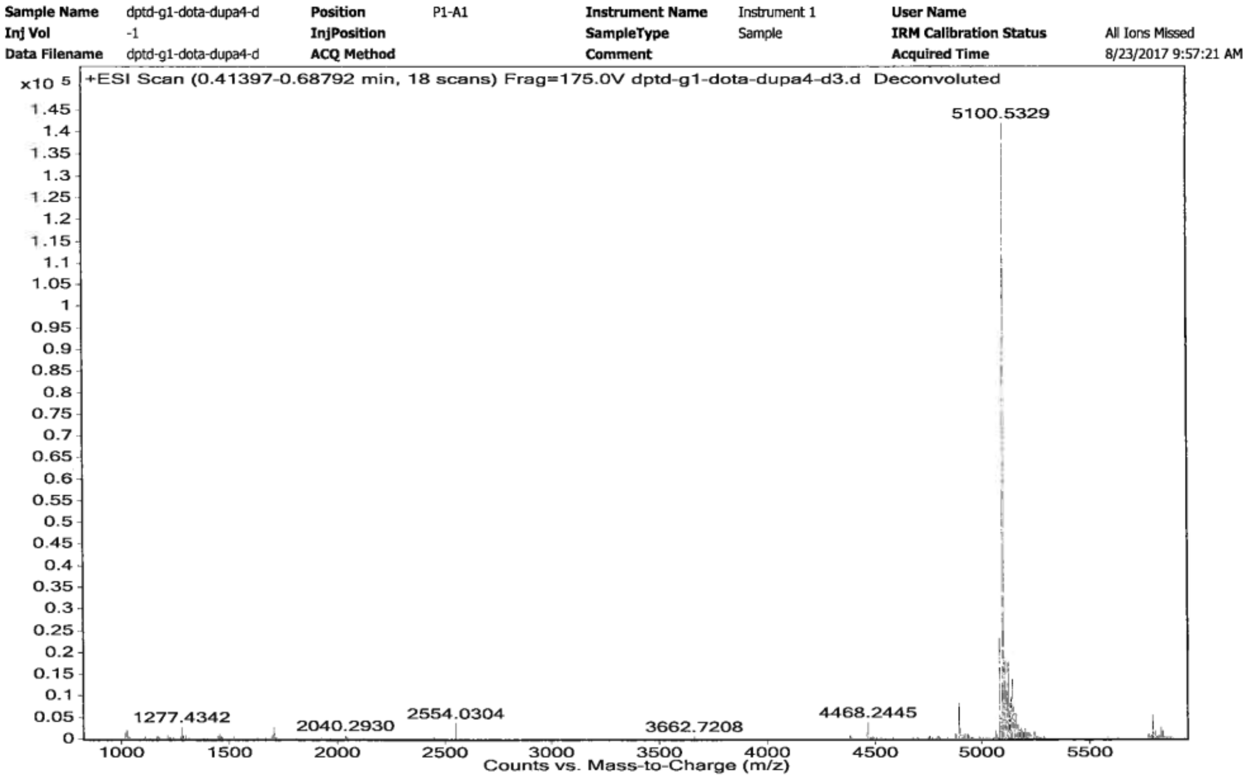

Figure S32. ESI-TOF mass spectrum of **G1-(DUPA)$_4$**.

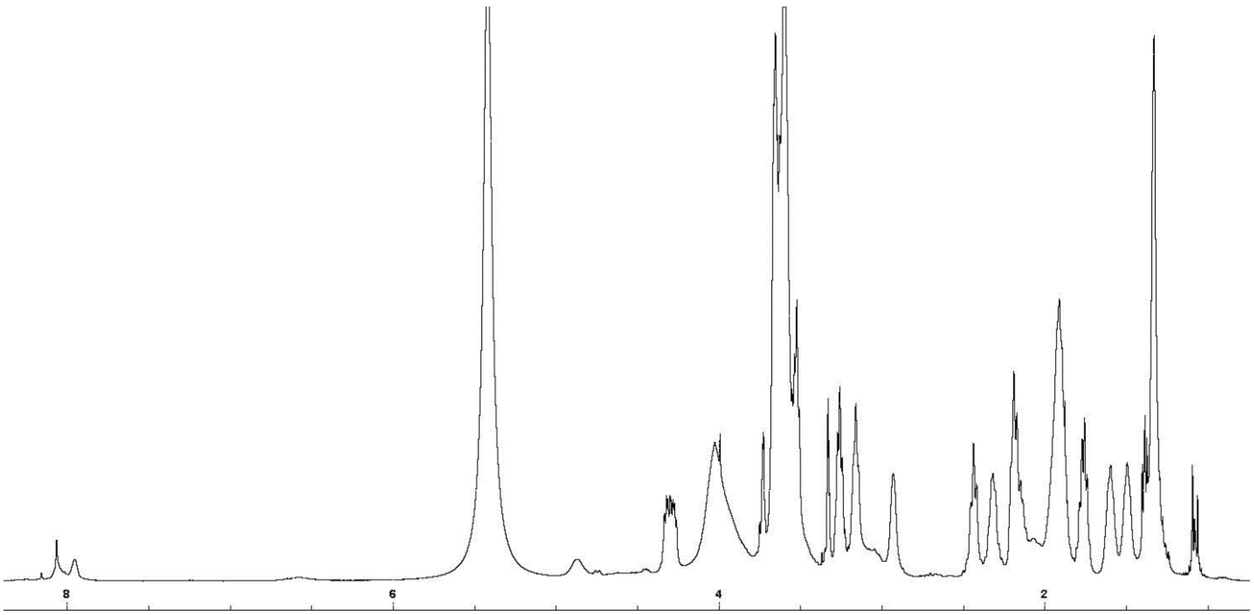

Figure S33. ^{1}H NMR spectrum of **G3-(DUPA)$_{16}$** (400 MHz, CD_3OD).

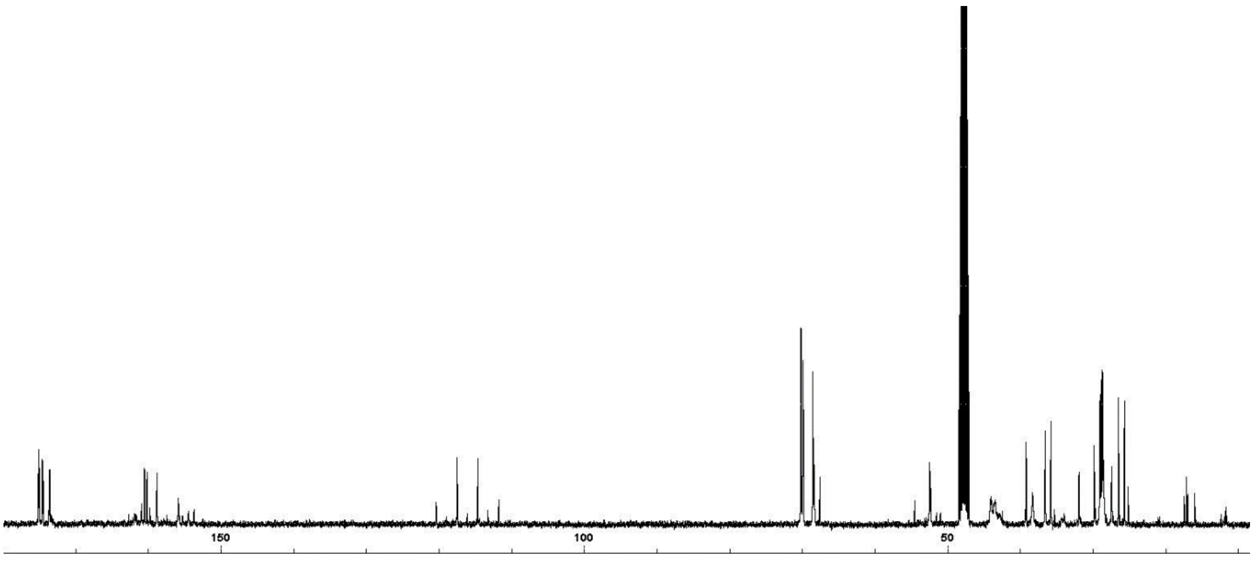

Figure S34. ^{13}C NMR spectrum of **G3-(DUPA)$_{16}$** (100 MHz, CD_3OD).

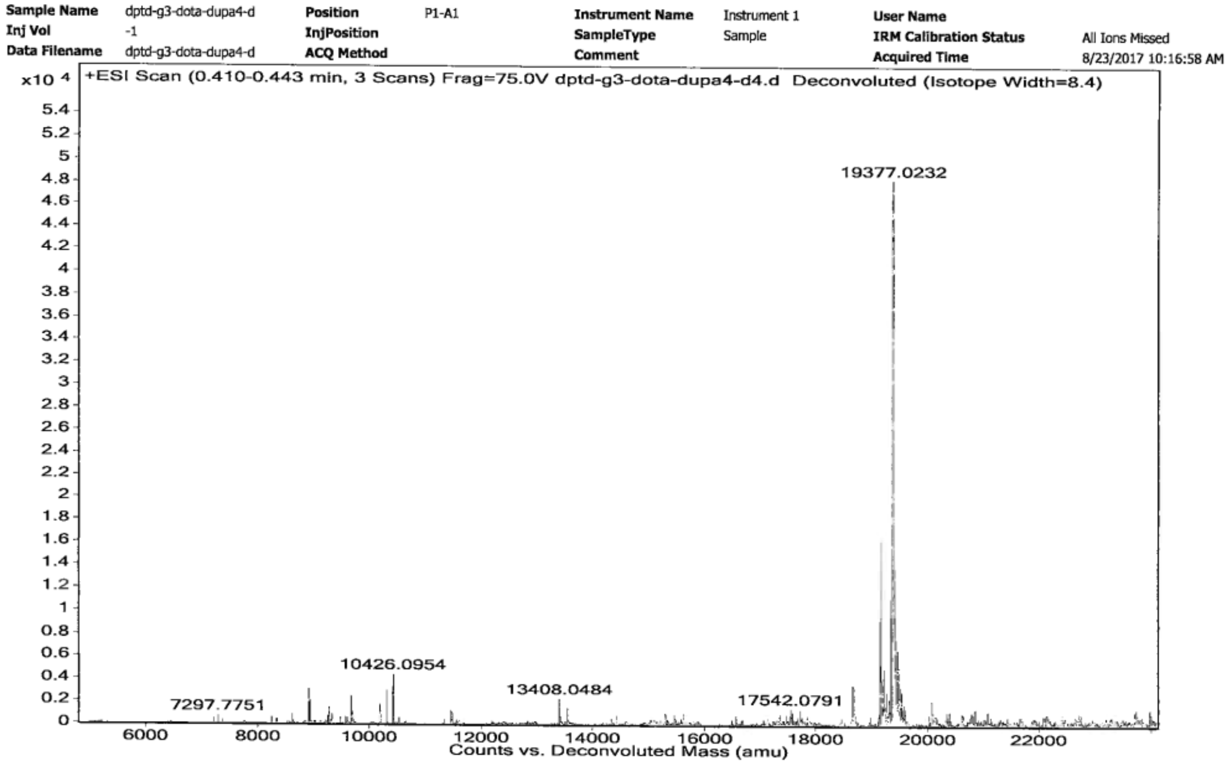

Figure S35. ESI-TOF mass spectrum of **G3-(DUPA)$_{16}$**.

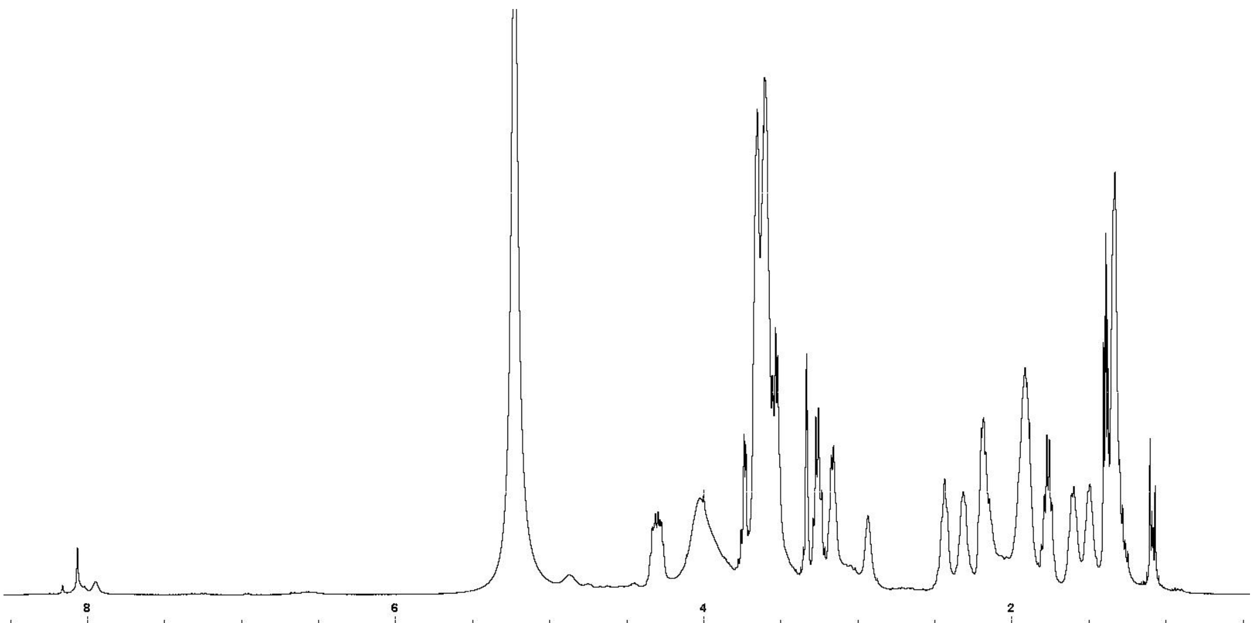

Figure S36. ^{1}H NMR spectrum of **G5-(DUPA)$_{64}$** (400 MHz, CD_3OD).

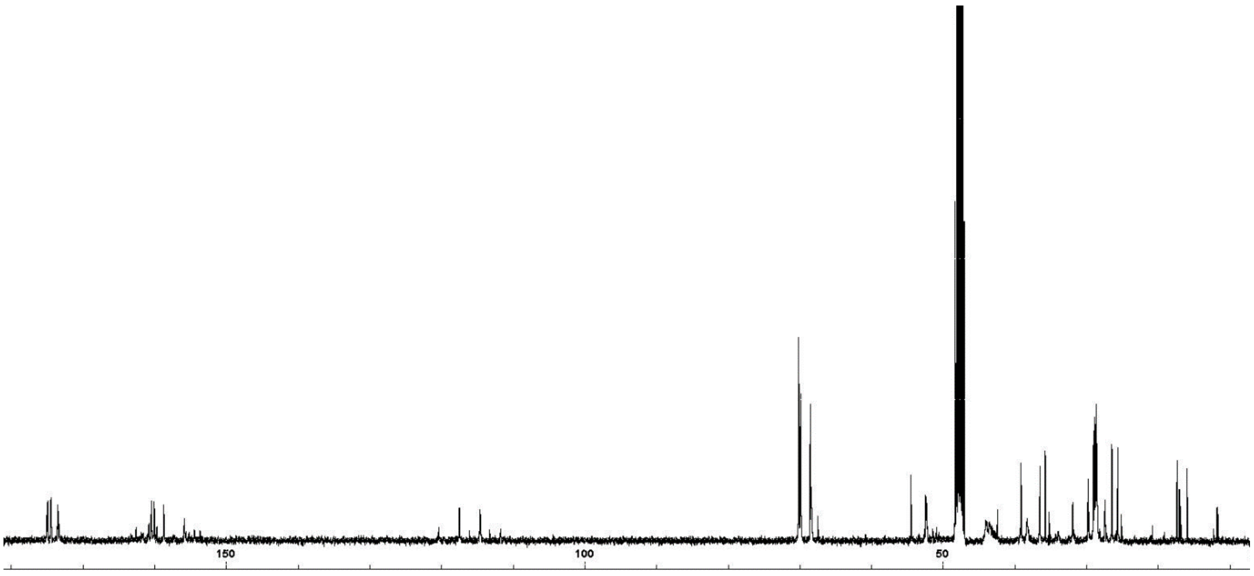

Figure S37. ^{13}C NMR spectrum of **G5-(DUPA)$_{64}$** (100 MHz, CD_3OD).

Radio-HPLC

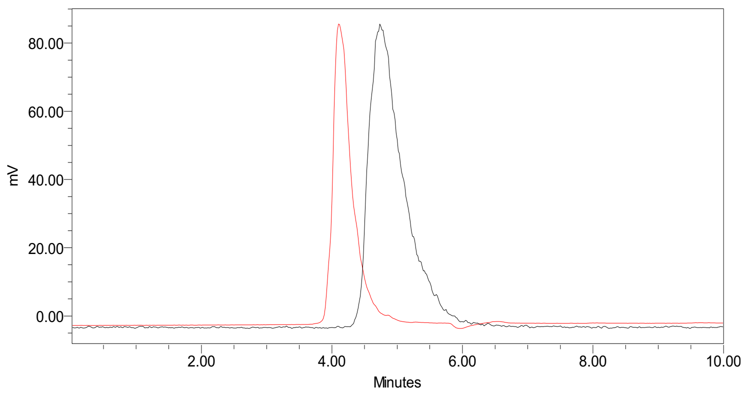

Figure S38. Radio-HPLC of ^{64}Cu-labeled **G1-(DUPA)$_4$** (red line: radioactivity detector, black line: UV-Vis detector).

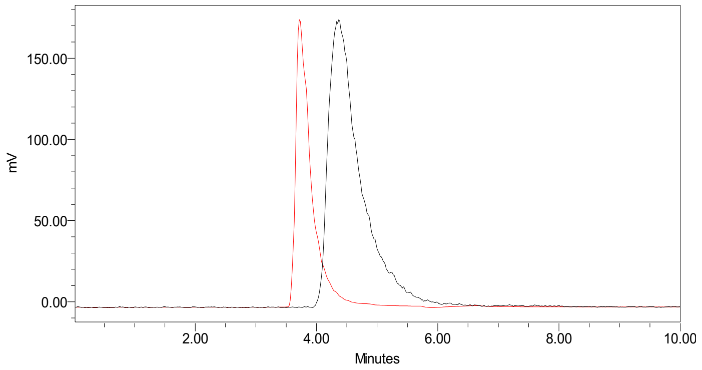

Figure S39. Radio-HPLC of ^{64}Cu-labeled **G3-(DUPA)$_{16}$** (red line: radioactivity detector, black line: UV-Vis detector).

.

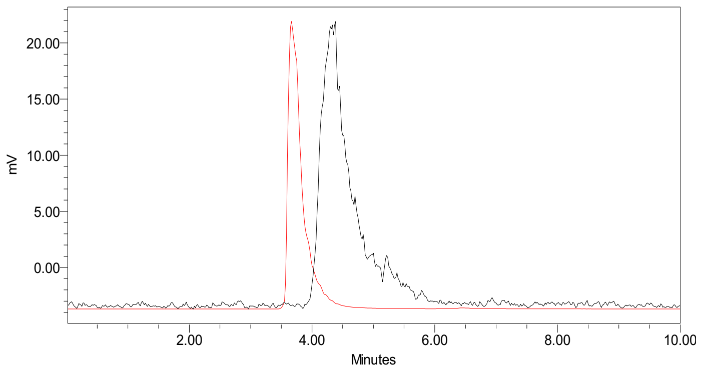

Figure S40. Radio-HPLC of ^{64}Cu-labeled **G5-(DUPA)$_{64}$** (red line: radioactivity detector, black line: UV-Vis detector).

Serum Stability Assay

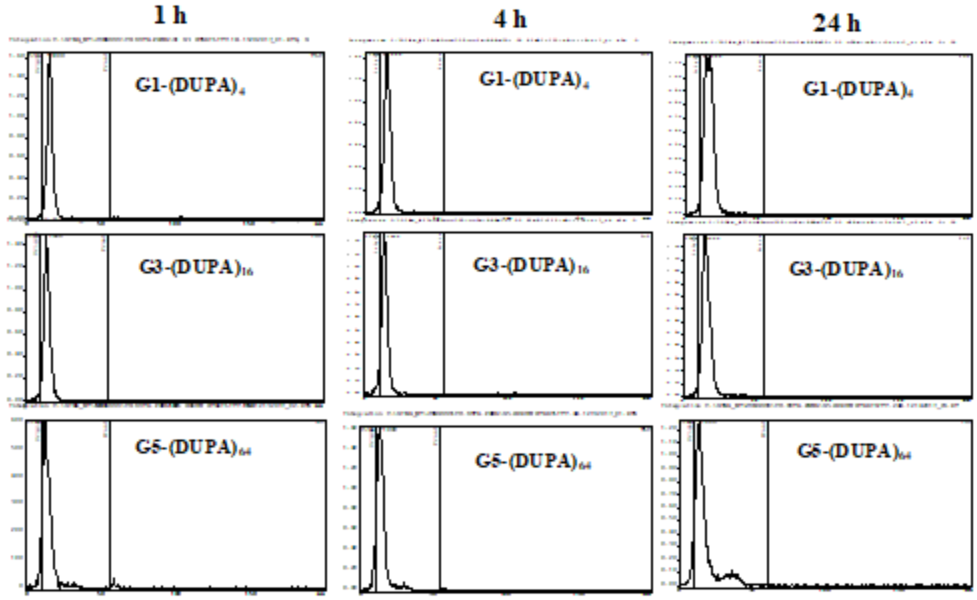

Figure S41. Serum stability assay of the ^{64}Cu-labeled dendrimers in rat serum (1 h, 4 h, and 24 h incubation).
